# Dopamine transporter oligomerization involves the scaffold domain, but spares the bundle domain

**DOI:** 10.1371/journal.pcbi.1006229

**Published:** 2018-06-06

**Authors:** Kumaresan Jayaraman, Alex N. Morley, Daniel Szöllősi, Tsjerk A. Wassenaar, Harald H. Sitte, Thomas Stockner

**Affiliations:** 1 Medical University of Vienna Center for Physiology and Pharmacology, Institute of Pharmacology, Vienna, Austria; 2 Groningen Biomolecular Sciences and Biotechnology Institute and Zernike Institute for Advanced Materials, University of Groningen, Groningen, The Netherlands; Icahn School of Medicine at Mount Sinai, UNITED STATES

## Abstract

The human dopamine transporter (hDAT) is located on presynaptic neurons, where it plays an essential role in limiting dopaminergic signaling by temporarily curtailing high neurotransmitter concentration through rapid re-uptake. Transport by hDAT is energized by transmembrane ionic gradients. Dysfunction of this transporter leads to disease states, such as Parkinson’s disease, bipolar disorder or depression. It has been shown that hDAT and other members of the monoamine transporter family exist in oligomeric forms at the plasma membrane. Several residues are known to be involved in oligomerization, but interaction interfaces, oligomer orientation and the quarternary arrangement in the plasma membrane remain poorly understood. Here we examine oligomeric forms of hDAT using a direct approach, by following dimerization of two randomly-oriented hDAT transporters in 512 independent simulations, each being 2 μs in length. We employed the DAFT (docking assay for transmembrane components) approach, which is an unbiased molecular dynamics simulation method to identify oligomers, their conformations and populations. The overall ensemble of a total of >1 ms simulation time revealed a limited number of symmetric and asymmetric dimers. The identified dimer interfaces include all residues known to be involved in dimerization. Importantly, we find that the surface of the bundle domain is largely excluded from engaging in dimeric interfaces. Such an interaction would typically lead to inhibition by stabilization of one conformation, while substrate transport relies on a large scale rotation between the inward-facing and the outward-facing state.

## Author summary

The human dopamine transporter efficiently removes the neurotransmitter dopamine from the synaptic cleft. Alteration of dopamine transporter function is associated with several neurological diseases, including mood disorders or attention-deficit hyperactivity disorder, but is also a major player in addiction and drug abuse. Functional studies have revealed that not only is transporter oligomerization involved in surface expression and endocytosis, but, more importantly, in reverse transport (efflux) of dopamine that is triggered by amphetamine-like drugs of abuse. Structural knowledge of transporter oligomerization is largely missing. We performed a large scale comprehensive computational study on transporter oligomerization to reveal dimer geometries and the residues involved in the interfaces. The dimer conformations we find in our dataset are fully consistent with all available experimental data in the literature, but also show novel interfaces. We further verified all dimer geometries by free energy calculations. Our results identified an unpredicted—but for the mechanism of substrate transport essential—property: the bundle domain, which moves during the transport cycle, is excluded from contributing to dimer interfaces, thereby allowing for unrestrained movements necessary to translocate substrates through the membrane.

## Introduction

The human dopamine transporter (hDAT) is a member of the monoamine transporter family [1], which also includes the transporters for serotonin (hSERT) and noradrenaline (hNET). Within the central nervous system, these transporters are localized to pre-synaptic neurons close to the synaptic junctions, but primarily outside the synapse [2]. Their role is to efficiently curtail neurotransmitter-mediated signaling via uptake of associated neurotransmitters into pre-synaptic neurons. These transporters utilize the sodium/chloride gradient across the plasma membrane to provide the driving force for transport [3]. Dysfunction in hDAT leads to severe neuronal disorders, such as Parkinson’s disease, attention-deficit hyperactivity disorder (ADHD), depression and schizophrenia [4,5]

The first crystal structure for this class of transporters became available for the bacterial homolog small amino acid transporter (LeuT) isolated from *Aquifex aeolicus* [6]. The *Drosophila* melanogaster dopamine transporter (dDAT) was the first eukaryotic member of the SLC6 family to be resolved by X-ray crystallography [7], followed more recently by crystallization of the human serotonin transporter (hSERT) [8]. LeuT was crystallized in three conformations [6,9,10], revealing that during the transport cycle the bundle domain (consisting of transmembrane helices TMH 1, 2, 6, 7) rotates by ~30° relative to the scaffold domain (TMH 3, 4, 8, 9.10, 12), thus anchoring the transporter to the membrane. TMH 5 and 11 are also assigned to the scaffold domain, though they bend during the conformational transition of the transport cycle. The similarity of these crystal structures confirmed that several findings can be conveyed from the bacterial LeuT to the human homologs, including transporter topology, conformational changes of the transport cycle [6,9–11] and the substrate binding site [12]. These structures also revealed important differences, as the human transporters do not share the dimeric form with LeuT, due to the conformation of transmembrane helix 12 (TMH12) [6,7]. TMH12 is central in the dimer interface of LeuT, but is remarkably different in dDAT and hSERT. Cross-linking experiments and Förster resonance energy transfer (FRET) measurements indicated the existence of higher-order oligomers of human monoamine transporters [13–20] in the plasma membrane. Functional studies revealed the importance of higher-order oligomers for their physiological function, and also revealed substrate-transport and inhibitor binding-dependent oligomerization [19,21–25]. Spectroscopic studies of hSERT showed that the transporter assumes a large distribution of oligomeric sizes [26,27]. The stability of the oligomer and protomer exchange rates were contingent on the presence of phosphatidylinositol-4,5-bisphosphate (PIP2) [28]. Monoamine transporters have mainly been simulated in their monomeric form, although LeuT-based dimers have also been examined [29,30]. Recently, protein docking predicted a single hDAT dimer conformation that included residue C306 in the interface, which was assessed using MD simulations [30] and site-directed mutagenesis studies.

In the current study, our key aim was to develop a comprehensive description of hDAT oligomerization by computational approaches. We hence characterized the interfaces of human DAT (hDAT) oligomers and identified the transmembrane helices and residues involved using a hDAT model based on the outward-facing dDAT crystal structure. Membrane properties, including lipid entropy, play a crucial role in the process of oligomerization. We have therefore used the unbiased DAFT (Docking assay for transmembrane components) [31] approach to directly follow the formation of hDAT dimers, starting from two randomly-oriented membrane inserted hDAT transporters. We observed the formation of four stable symmetric and four asymmetric dimer conformations. Importantly, we found only a very modest contribution of the bundle domain in the dimer interfaces, consistent with its role as a moving domain during the transport cycle.

## Results

**Fig 1:**
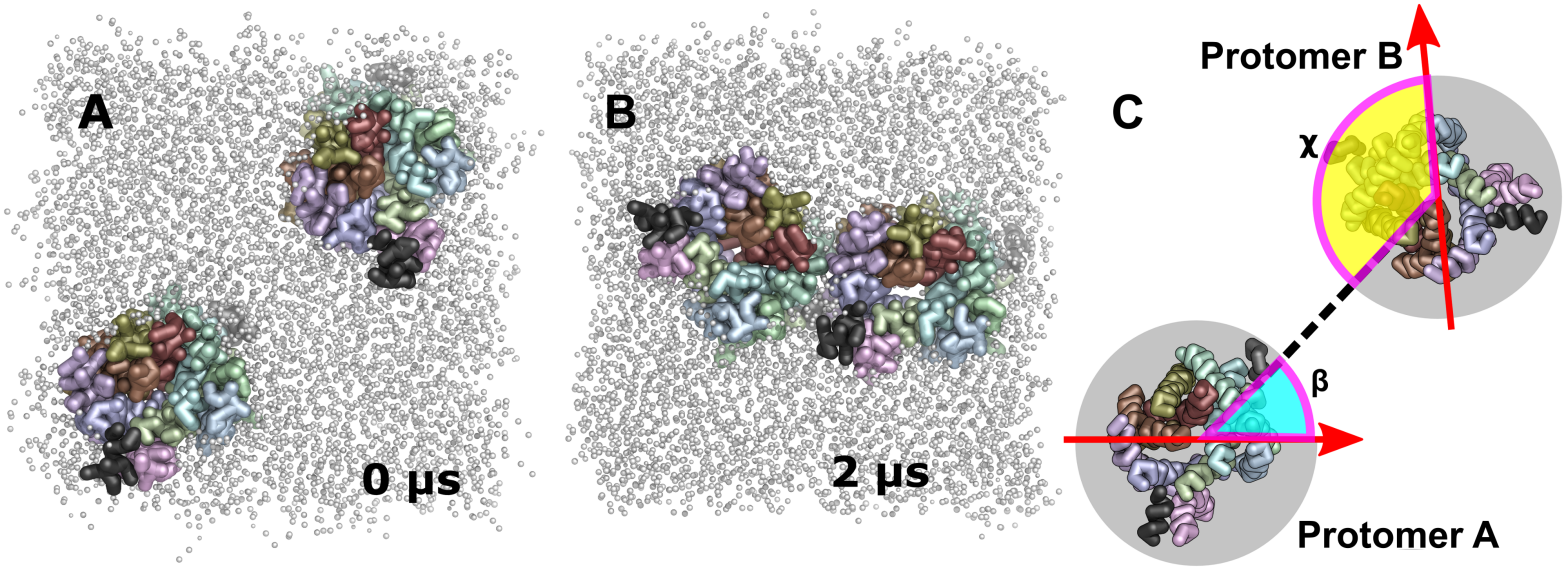
Schematic representations. Representative system showing randomly oriented A) starting (0 μs) and B) end (2 μs) structure in coarse-grained representation. For the protein, only the backbone of the transmembrane helices and the C-terminal helix are shown. The transporter is visualized from the intracellular side. C) Schematic representation to illustrate the quantification of relative hDAT dimer orientation. The transporter is visualized from the intracellular side. The red arrow represents the reference vector or direction within the frame of individual hDAT transporter. It is oriented roughly towards TMH4 and located within the plane of the membrane. The angle β (cyan area) quantifies the center of mass position of protomer B relative to the reference frame in protomer A by measuring the angle between the reference vector of Protomer A and the line connecting the center of masses of both protomers. The angle χ (yellow area) determines the center of mass position of protomer A relative to the reference frame in protomer B by measuring the angle between the reference vector of Protomer B and the line connecting the center of masses of both protomers.

Neurotransmitter transporters are known to oligomerize in the plasma membrane [32]. It was shown that the oligomeric state modulates transporter function [22], but structural knowledge is largely missing. Only a few residues have been found to most likely reside within protomer interfaces [13,14,17,21]. The aim of this study was to observe dimer conformations and to identify transmembrane helices and residues involved in hDAT dimerization. In this study we used an unbiased computational approach [31] (see Material and methods for detailed description), in which we repeatedly embedded two hDAT transporter molecules in random relative orientation in a palmitoyl-oleoyl-phosphatidyl-choline (POPC) membrane bilayer and simulated each system for 2 μs (Fig 1). This process was repeated 512 times to obtain an ensemble of structure and trajectories (total simulation time of over 1 ms) that is large enough to be representative for hDAT oligomerization.

### Relative protein orientation

Relative orientation of the two protomers in the dimer was assessed through the introduction of an internal coordinate system, as shown in Fig 1C. We first defined an orientation vector as the reference vector (red arrow) within the frame of each hDAT molecule. The position (β) of protomer B relative to protomer A is measured as the angle between the reference vector and the vector connecting the center of mass of the two hDAT monomers. In addition to the orientation described in the β angle, the second hDAT (protomer B) molecule can also rotate around it own axis. Therefore, we introduced a second angle (χ) that measures the orientation of the reference vector of protomer B relative to the line connecting the center of mass of the two hDAT molecules.

**Fig 2:**
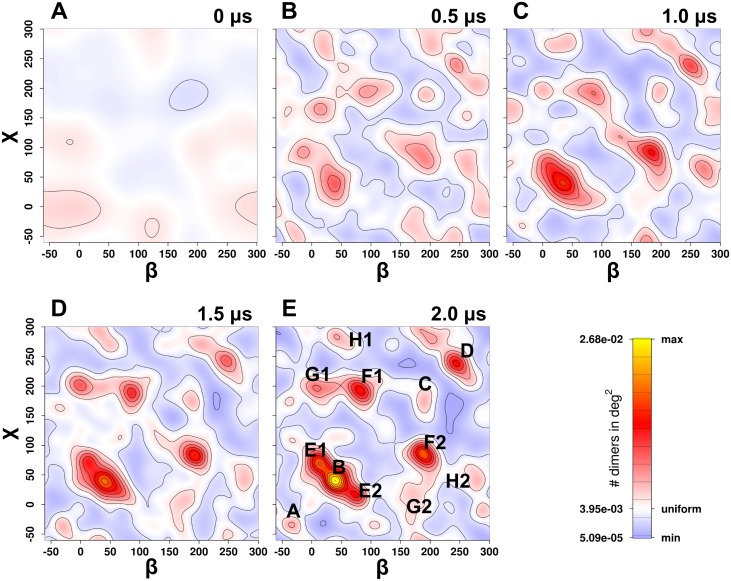
Orientation plot. Time evolution of dimer orientation (panel A–E) shown at 0.0, 0.5, 1.0, 1.5 and 2.0 μs, respectively. Axis labels β and χ represent the relative orientation of the hDAT dimer as outlined in Fig 1. The area of blue color highlights orientations which are less frequent than expected with a random orientation, red to yellow colors are indicative of high dimer density. The clusters formed are labeled in panel E: the clusters labeled as A-D on the diagonal are symmetric. Each off-diagonal cluster is present twice, because they are symmetry-related and represent the same conformation, as protomer A and protomer B are interchangeable in a homodimeric structure. These are labeled as E1-H1 above the diagonal and E2-H2 below the diagonal.

The orientation plot (Fig 2) summarizes across the ensemble the relative orientation of the two protomers using these two angles (β and χ). The orientation plot therefore reflects the frequency with which each orientation was observed, while not averaging over the symmetry-related orientation of the homodimers. The series of plots in Fig 2 shows the time evolution of orientations at 0.0 μs, 0.5 μs, 1.0 μs, 1.5 μs and 2.0 μs. Over time an enrichment of a few prominent clusters or orientations became apparent, visible as red-to-yellow regions, while the frequency of observing other conformations decreased, which are indicated by blue colored areas, in which the dimer probability is below average.

The orientation plot is symmetric with respect to the diagonal once converged, because we measure the orientation of homodimers. Clusters on the diagonal represent symmetric dimers, while all off-diagonal dimers are asymmetric, differing in the helices in the shared interfaces. We did not impose symmetry in the analysis, but rather used it as a measure of convergence. The inherent symmetry of the orientation plot requires that at full convergence all off-diagonal peaks must be present in both symmetry-related positions and must be sampled with the same frequency. The plot at 2.0 μs was remarkably symmetric, indicative of an almost fully converged dataset in which the appearance of additional conformations at even longer simulation times is unlikely. The time evolution of the degree of symmetry of the four off-diagonal clusters E-H showed that the ratio converges from 1:3 at 0.5 μs to 1:1.2 at 2.0 μs. The ratios were estimated by calculating the difference between the number of systems in the four clusters above the diagonal over the number of systems in the four clusters below the diagonal. The second applied measure of convergence was the time evolution of the potential energy of hDAT-hDAT interactions (S1 Fig). The plot showed that interacting dimers were present in large numbers and the profile is leveling off. Exchange between dimer conformations with the same interaction energy cannot be detected by the interaction energy plot, but would remain visible in the orientation plot. The plateau in the interaction energy plot is reached once the number of interacting dimers in the full ensemble has equilibrated and the populations of weaker and stronger interacting dimers no longer change. Experimental data for the parolog human SERT have shown that ~35% of all hSERT transporter are monomeric in the endoplasmatic reticulum (ER) membrane as well as in the plasma membrane [27,28]. The ER membrane is similar to our bilayer in that it mainly consists of phosphatidyl lipids, while being almost devoid of cholesterol and free of PIP2. Given the similarity between hSERT and hDAT we should also not expect that all hDAT transporters in the ensemble interact and form dimers. When comparing frequencies between symmetric and asymmetric dimers, the two symmetry-related off-diagonal conformations of an asymmetric cluster must be added together, while for the diagonal clusters these are already integrated, because of overlapping on the diagonal in the plot. In total, we found four symmetry-related conformations on the diagonal (cluster A-D) and four asymmetric conformations not on the diagonal (cluster E-H) were observed (See S1 Table for number cluster members).

### Conformational preference

**Fig 3:**
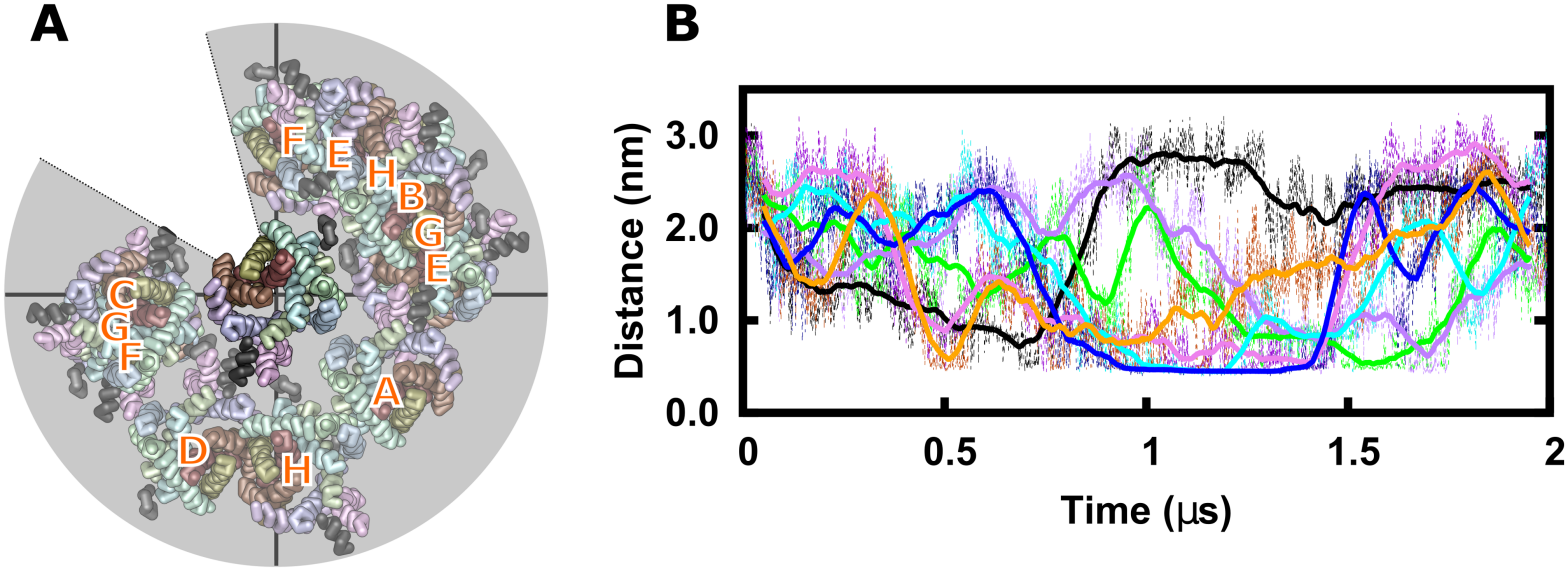
Dominant dimer orientations. A) Relative orientation of representative dimers extracted from each cluster after fitting to the first protomer. The four transmembrane helices of the bundle domain (TMH1, 2, 6, and 7) are highlighted in surface rendering. B) Minimum distance between protomers of all dimers from the entire ensemble that showed contacts which included the bundle domain.

The orientation plot showed that hDAT dimers clustered into a limited number of conformations. To set these into a structural perspective, we fitted all final structures (512 structures) to the first protomer and analyzed the position of the second protomer. The distribution of the second protomer was, as expected, not uniform throughout the ensemble (S2 Fig). In Fig 3A we show an overlay using one representative dimer per cluster (Fig 2D). All dimers were fitted to protomer A to place all 8 clusters into a single reference frame. Position and orientation of protomer B therefore shows its relative arrangement in the hDAT dimers. Importantly, this overlay revealed that possible dimer interfaces included a large part of the membrane-exposed hDAT surface, enclosing the transmembrane helices of the scaffold domain, but also highlighted a prominent exception, which was adjacent to the bundle domain. A comparison with the orientation plot (Fig 2) revealed that the frequency of finding dimers that included the bundle domain within the interface (corresponding to the region of ~105–150 °) was decreasing over time.

We then screened all trajectories to investigate if any dimer was formed in which the bundle domain was central for the dimer interface at any time-point throughout the 2 μs long simulations. In a condition of complete random encounters, we should have expected 20–40 contacts, but we found only 7 such trajectories (1.4% of all simulations). In all seven simulations, these dimers were unstable and separated within 0.5 μs (Fig 3B).

### Dimer interfaces

**Fig 4:**
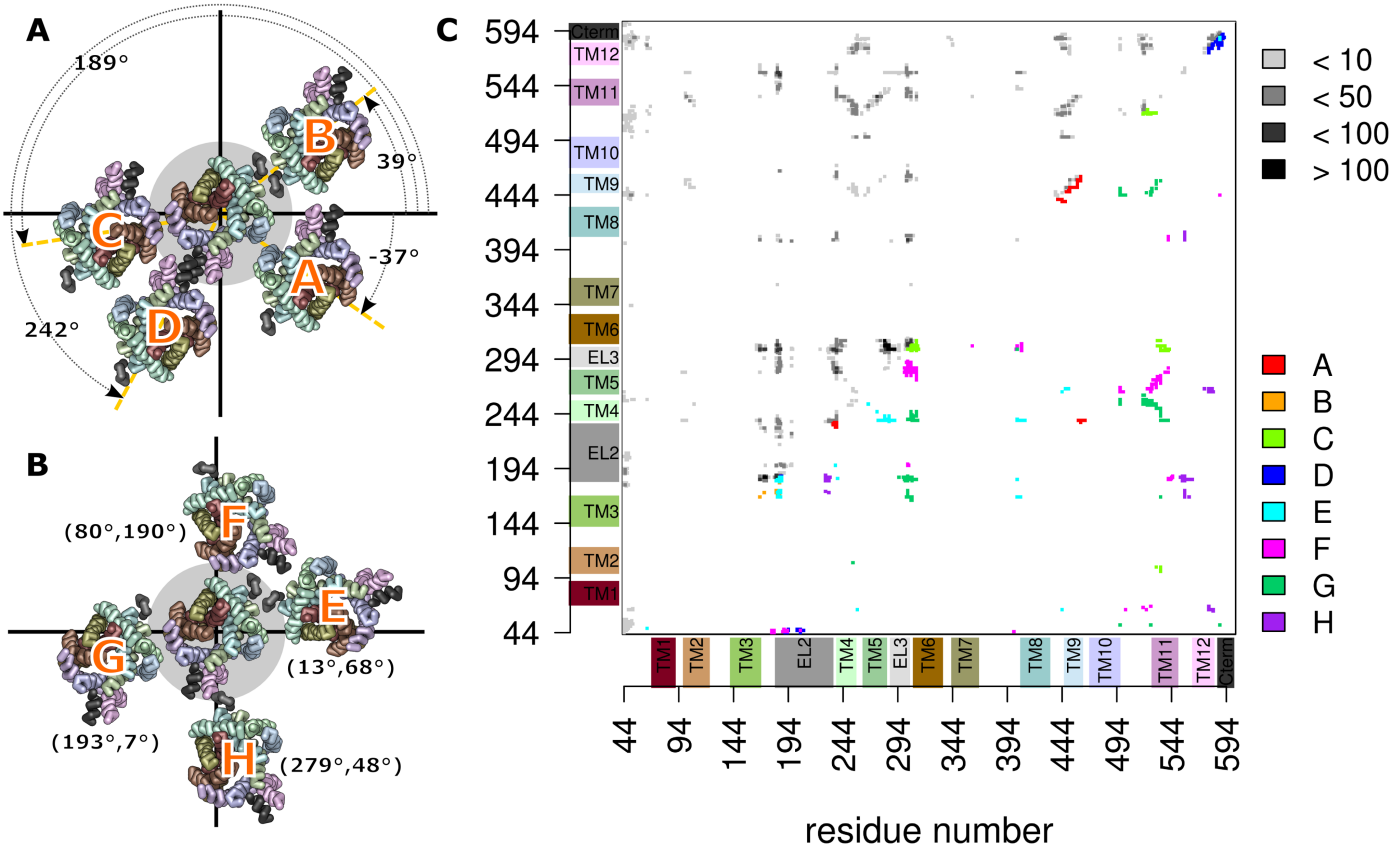
Dimer interactions. Representative dimer structures from each cluster of the A) symmetric and B) asymmetric, fitted to the first protomer. Only transmembrane helices, C-terminal helix and EL2 are shown for clarity. C) Quantification of helices and loops found within the dimer interfaces. Both axes are labeled with residue numbers. The transmembrane helices are indicated next to the axes, and we use the same color as in the all hDAT structure images. The plot shows the number of interactions for every residue pair using 5 frames of the last 100 ns of each DAFT trajectory of the complete dataset of 512 simulations. The number of observations is indicated in gray-scale colors. Below the diagonal are the residue-residue interactions of the dimers associated with one of the clusters A-H, colored according to the cluster.

The orientation plot showed the formation of 8 distinct clusters. One representative dimer was extracted from every cluster, converted into fine-grained representation and simulated for 100 ns to test their stability at the all atom resolution of classical force fields. These systems proved to be stable, confirming that the structure obtained through the DAFT workflow at the coarse-grained representation are stable low-energy conformations (S3 Fig). Fig 4A and 4B show representative structures for every cluster, whereby all dimers are fitted to protomer A. The position of protomer B is thereby shown (and indicated in degrees) using the internal coordinate system as references, as is also used in Fig 2. Comparison between Fig 4A and 4B shows that the binding geometry of some clusters like cluster C and G have mutually exclusive conformations, while clusters A and G could in principle contemporaneously form hDAT-hDAT dimers, leading to higher oligomeric structures.

Analysis of the transmembrane helices and loops within the interfaces is summarized in Fig 4C. The axes of Fig 4C are labeled by residue numbers; transmembrane helices and (frequently observed) loops are indicated next to the axes, colored according to the colors used for all DAT structures. Fig 4C shows a contact heat map at single residue resolution, integrated over the last 100 ns of each trajectory. Contact frequencies between residues are shown in the triangle above the diagonal coded in gray scale. It shows that some interactions are very frequent, indicating that these interactions could be essential for dimer stability. We find that transmembrane helices TMH1, 2, and 7 are completely absent in any interface. TMH6 can be found in the interface, although it is limited to interactions of residues of the first helical turn.

The lower panels color residue contacts according to association to one of the clusters. Overall, most contact on or close to the diagonal are from symmetric dimers. A per residue analysis (S4 Fig) shows interactions and interaction frequency for all dimers associated with one of the clusters. The symmetric dimers include less transmembrane helices within the interface regions than the asymmetric dimers. This was expected, because in the symmetric assembly the same regions are present on both protomers. On average, the interfaces of the symmetric dimers consisted of 1–2 transmembrane helices or loops on each protomer surface. We can infer from experimental data on the hSERT [28] that showed exchange rates of protomers on a time scale of minutes, that dimer association and dissociation are slow processes. Interestingly, the exchange rate was comparable between the cholesterol-free ER membrane and the cholesterol-containing plasma membrane, if devoid of PIP2, indicating that the exchange rate is not very sensitive to the membrane composition. Sequential residues along a helix change orientation by ~100° relative to the main helix axis. The interaction of helices therefore follows an alternating pattern, which is visible in Fig 4C as a strand that is oriented parallel to the diagonal (for parallel-oriented helices) or normal to the diagonal (for antiparallel-oriented helices), while the interacting residues show the typical heptad repeat pattern (S4 Fig). The length for the pattern is representative for the extent of helix-helix interactions.

### Symmetric dimers

**Fig 5:**
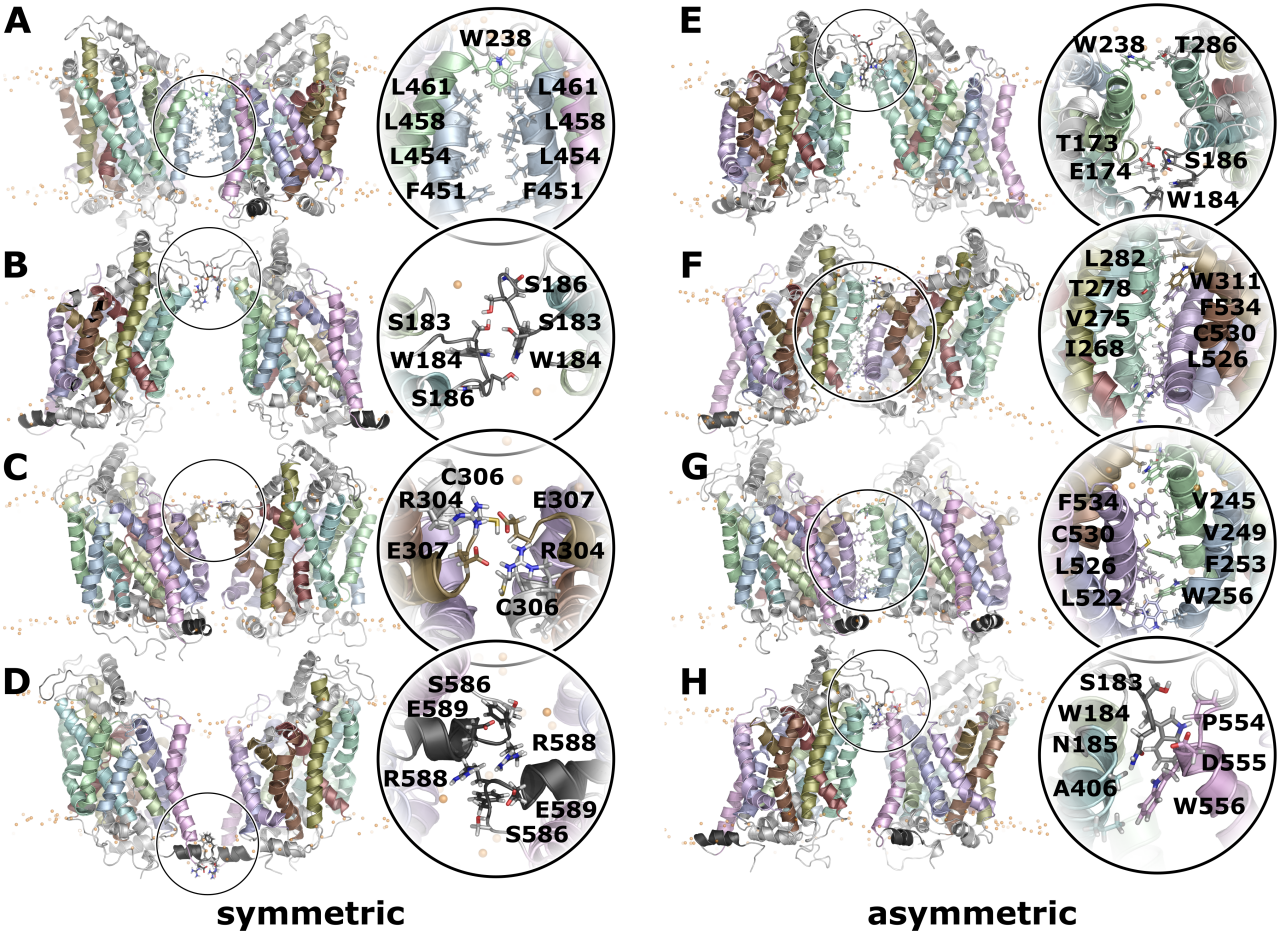
Representative dimer structures. A side view of a representative dimer structure is shown for each cluster (A-H). The insert zooms into the interface region from the same membrane side view or from the membrane plane. Residue side chains in contact with the second protomer are highlighted.

The symmetric interfaces of clusters B and D were dominated by interactions of residues outside transmembrane helices (Fig 5). Cluster B was mainly stabilized by interactions between the EL2 loops at the extracellular site, whereby the highly-conserved residues W184 and N185 were found in the core of the interface. It was shown experimentally that a mutation of W184 leads to a loss of surface expression [33]. Possible explanations might be a folding defect, which would lead to transporter degradation or a lack of transporter dimerization, which is required for ER export. Several lipids filled the space between the protomers. A similar lipid-bridged dimer was observed for cluster D, in which the protomer interactions were dominated by interaction between the C-terminal helices at the intracellular site. The dimer interface was stabilized by the salt bridge between R588 and E589 across the dimer interface, and further stabilized by additional interactions between aromatic residues on the C-terminal helix. The configurations of clusters B and D shared an additional property. The EL2 loop and the C-terminal helix were immersed in the membrane, and thus strongly interacted with the lipid headgroup region. In addition to direct interactions, the immersion perturbed the membrane bilayer, which could contribute to the attractive dimer-stabilizing forces due to membrane deformation.

In contrast, the symmetric interfaces of clusters A and C established transmembrane contacts (Fig 5). In cluster A, these comprised the entire TMH9, while TMH4 contributed mainly at its extracellular side. The geometry of the interface in cluster C was less specific, as TMH6 and TMH11 were within the interface in only a subset of the dimers. Common to both clusters was the fact that they included the cysteine residues C243 and C306, which have been shown by cross-linking experiments to be part of interfaces [13,14]. Cluster C showed dimers, which were stabilized by symmetric inter-molecular salt bridges between R304 and E307. These interactions oriented the two C306 residues to face each other at cross-linking distance. The conformation in cluster C was mainly stabilized by this pair of salt bridges, as the shared surface is otherwise small, leaving space to allow for dimer plasticity, mobility and dynamics.

### Asymmetric dimers

The off-diagonal clusters in the orientation plot are asymmetric and therefore share different interfaces (Fig 5). The orientation plot (Fig 2) showed four main off-diagonal clusters and also revealed that the interface on one protomer was able to interact with more than one interface on the second protomer. Cluster E1/2 has several properties in common with cluster B, including the EL2 loop that is prominently present in the interface and the lack of extensive contacts in the hydrophobic core of the membrane. The EL2 loop interacted with TMH3 and 4 on the first protomer and TMH5 on the second. Cluster H1/2 shared the EL2 loop as part of the interface, but the orientation of the second protomer was remarkably different from cluster E2/3, as the contacting interface was mainly TMH12, though limited to its extracellular side. The A559V mutation is located within the extracellular region of TMH12 and is associated with Autism spectrum disorders (ASD) [34,35], caused by trafficking and functional alterations, while the P554L mutant, located in EL5, is associated with the Dopamine Transporter Deficiency Syndrome (DTDS) [36] and has been described as intracellularly retained.

The dimer clusters (F1/2 and G1/2) formed an extensive interface throughout the transmembrane region. Both clusters shared TMH11 in their interface, often including contacts to EL3. TMH11 has been reported to be located in dimerization interfaces [17] of human monoamine transporters. The main helix interfacing with TMH11 was TMH5 in cluster F1/2 and TMH4 in cluster G1/2. This change in relative orientation is achieve by a ~60° rotation of the second protomer. Cluster G1/2 is of particular interest, because residue C243 and C306 were found at cross-linking distance from each other. Cross-linking experiments showed that hDAT can form asymmetric trimer and tetramers [14] through a chemical bond established between C243 and C306.

### Potential of Mean Force profiles

**Fig 6:**
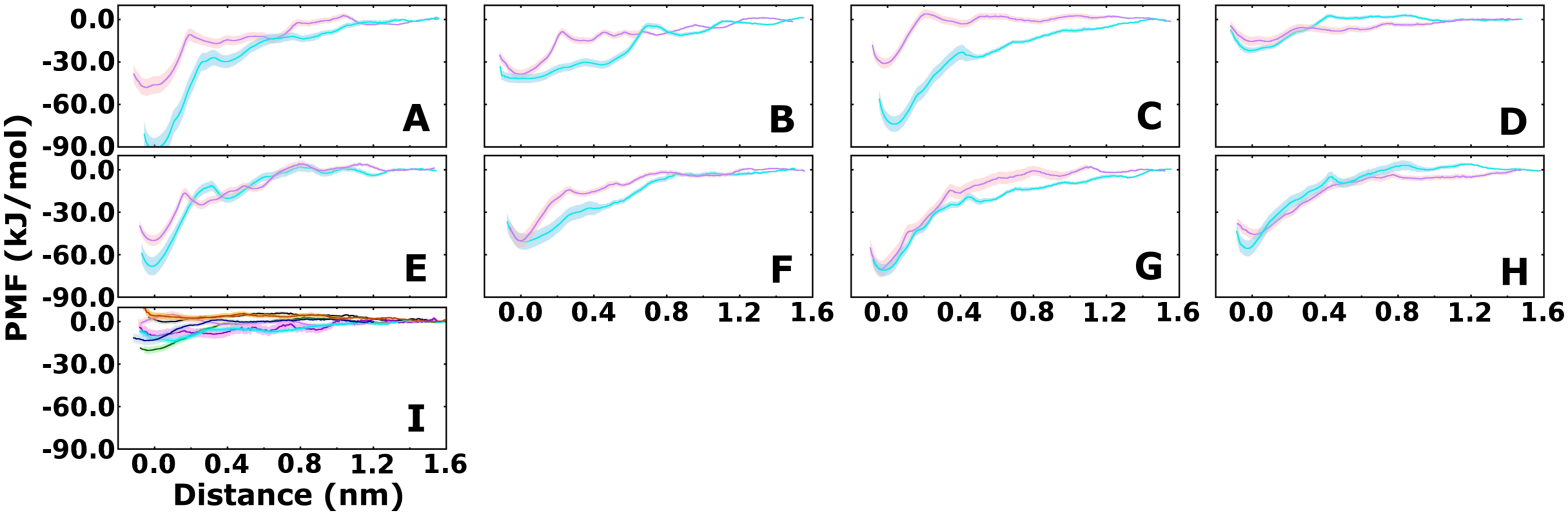
Potential of Mean Force. Panels A-H show PMF profiles of protomer separation of two dimers per cluster, revealing the stabilization energy of each dimer. Panel I shows the essentially flat PMF profiles for every transient dimer that dominantly interacted with the bundle domain during the DAFT simulations (See Fig 3B). A PMF profile for the respective system was calculated starting from the frame of the DAFT simulations with the smallest hDAT-hDAT distance. The same color code was applied as used in Fig 3B.

We quantified the stability of observed dimers by determining Potential of Mean Force (PMF) profiles for dimer dissociation of two representative dimers per cluster (Fig 6) by applying the same CG system representation as used for the DAFT simulations. The starting structures of all systems are shown in S5 and S6 Figs. In the first step, the two protomers were pulled apart by steered MD (SMD) simulations in a 100 ns long trajectory, while restraining the relative orientation using the enforced rotation module. The applied pulling velocity (0.025 nm/ns) was set to be in the range of the fastest diffusion observed in the unbiased simulations. The rationale for selecting such a slow pulling rate was to use a velocity that is compatible with normal diffusion in the membrane in order to obtain SMD trajectories that minimally perturb the membrane. In the second step we extracted equidistant structures at 0.1 nm separation over 1.6 nm and carried out PMF calculations using umbrella sampling. Additional conformations with 0.025 nm increments over the first 0.4 nm were added in the initial rising part of the PMF to improve sampling and achieve sufficient histogram overlaps. Overall, the PMF profiles of dimers extracted from all clusters showed qualitatively similar profiles, with a dimer-stabilizing potential between 30 and 90 kJ·mol^-1^ (Fig 6). S2 Table reports the integrals of each PMF profile. The error bars reflect the variability observed in the PMF calculations and represent lower boundaries, because additional variability which might only appear over a much longer timescale is not sampled in the umbrella window calculations. The restraining forces of the enforced rotation module applied to prevent translation (S7 Fig) and rotation (S8 Fig), showed a Gaussian-like distribution, which indicates that the restraints did not mask any hidden energy gradient or forces. To provide a better estimate of the variability of the PMF profiles, we calculated two PMF profiles per cluster, using two different hDAT dimers as starting structures. The profiles of clusters D-H showed limited difference between the two profiles, suggesting that these are representative profiles. The profiles for the clusters A, B and C showed a large difference between the two profiles. The very large energy difference between the dimeric and the monomeric state of one profile of cluster A indicates that this profile might be overly attractive. The difference between the two profiles of cluster B and C are more likely a consequence of differences in the starting structures, which were selected according to the centrality of each cluster, but differed in the details of their interfaces (S5 and S6 Figs). The dimers of clusters B, D, but also F, showed weaker and broader interaction profiles of 20–50 kJ·mol^-1^. The dimers with a large transmembrane interface exhibited especially strong and short-range interaction profiles, showing 60–75 kJ·mol^-1^ of stabilizing energy. The short-range nature of these profiles indicate that they are dominated by van der Waals interaction between transmembrane helix side chains, while the broader profile indicates an important role of lipids and/or the changes on the intracellular and extracellular loops. Of particular importance were the details of loop interactions across the dimer interface as obtained from the final structure of the DAFT simulations. The broader minima were associated with the sliding of residues from the entangled opposing loops during the initial phase of separation.

The PMF profiles were strikingly different for the 7 dimers that formed transient interactions involving the bundle domain (Figs 3B and 6I). From every unbiased trajectory we extracted the frame with the smallest dimer-dimer distance as a starting point for the PMF calculations. All seven profiles showed a flat energy landscape, revealing that these structures were not held together by any stabilizing interactions, therefore transient and short-lived.

### Electrostatic fields

**Fig 7:**
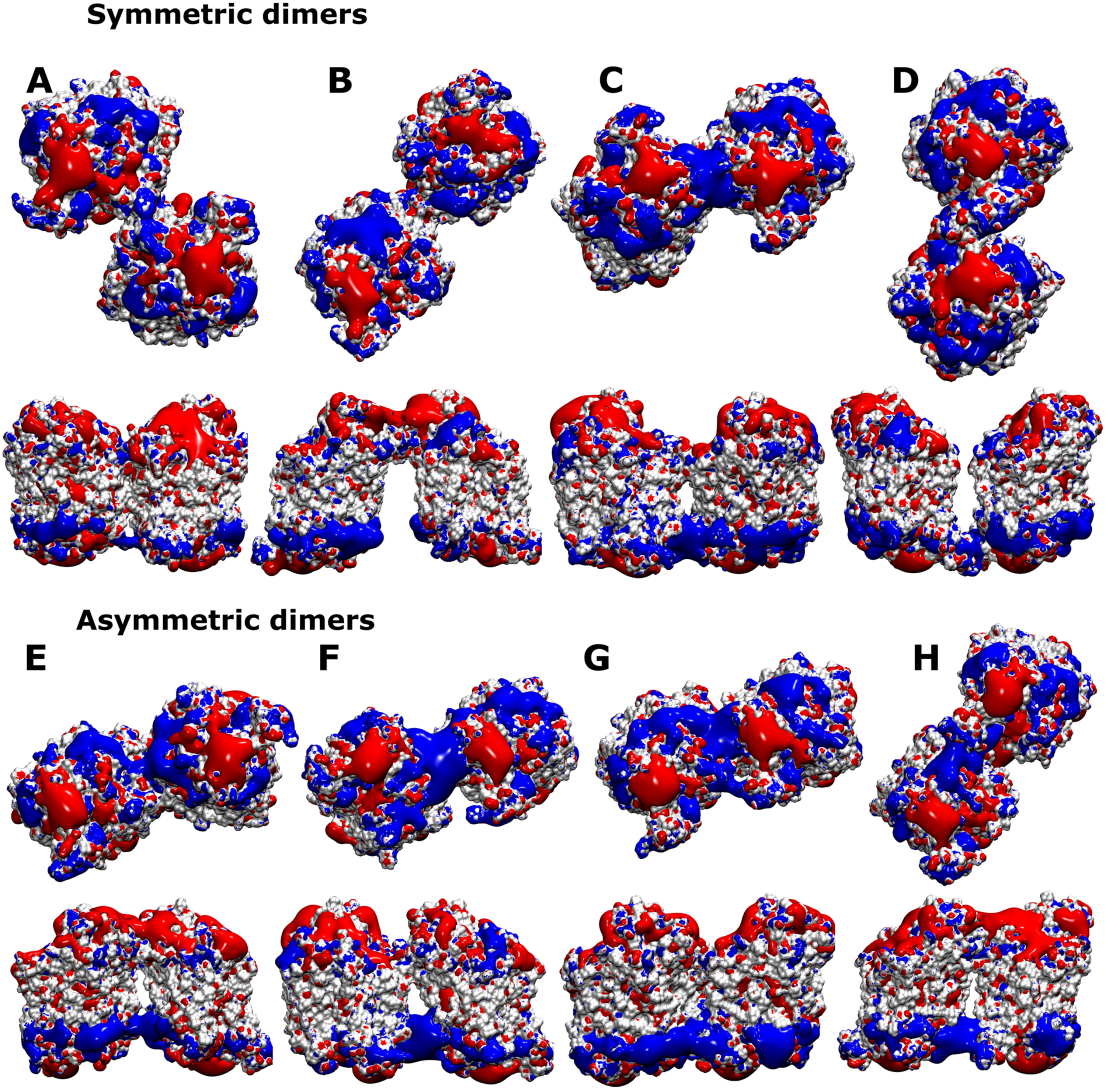
Electrostatic fields. A cytosolic view and a side view is shown for a representative hDAT dimer structure for each cluster, indicated by the labels A-H. The hDAT surfaces are shown in white, while fields generated are shown in blue (positive potential) and red (negative potential). Potential surfaces are drawn at 2eV.

Binding of the negatively-charged lipid PIP2 was shown for hSERT to affect oligomerization [28,37,38]. The protomers in each oligomer exchanged with a timescale of minutes in both the plasma membrane (if depleted of PIP2) and the ER. The presence of PIP2 in the plasma membrane blocks this exchange process and kinetically traps the oligomer in its state at the plasma membrane. PIP2 carries a charge of -5. We thus assumed that binding is dominated by electrostatic interactions and calculated the electrostatic potential for each cluster (Fig 7) using apbs [39]. A representative structure for each cluster was converted from the coarse-grained representation to a fine-grained all-atom structure using the back-mapping module [40] by applying two steps of energy minimization and four steps of system relaxation. Visualization of the potential isosurfaces at 2 eV revealed potential PIP2 binding sites as areas of a positive electrostatic potential extending into the membrane. A large positive electrostatic field reaching into the membrane was generated by three clusters: the symmetric cluster C, which includes C306 in its interface at the extracellular site, the asymmetric clusters F1/2 and G1/2, which both showed extensive contacts across the lipid bilayer. The respective fields were generated by the positively-charged lysine and arginine residues of IL5 for the symmetric cluster C, by residues in IL3, IL5 and the N-terminus for cluster F1/2 and by residues in IL2, IL4 and IL5 in cluster G1/2. These fields extended into the headgroup region of the lipid bilayer, therefore pre-disposing these conformations for interactions with negatively-charged lipid such as phosphatidylserine or PIP2.

## Discussion

The physiological role of the hDAT is to rapidly remove the previously released dopamine by uptake in the pre-synaptic neuron, which leads to a fast drop of dopamine concentration. Oligomerization of human monoamine transporters is involved in transporter function at multiple levels: surface expression requires transporter dimerization to pass quality control at the ER exit sites for loading into COPII vesicles [41]. Transporter function seems to be modulated by oligomerization, including substrate transport activity and amphetamine-elicited neurotransmitter efflux by reverse transport through the transporter [19,23,24] as well as endocytosis from the plasma membrane [42]. Also, some evidence of cooperativity between transporter protomers has been reported in the literature [22,25,43]. Early studies on transporter oligomerization have identified C243 and C306 [13,14] in hDAT to be involved in oligomer formation. These two residues are positioned at opposite sites on the transporter surface, i.e. C243 is located within TMH4, while C306 is found towards the end of EL3. It was therefore conceivable that large oligomer structures of theoretically infinite size could form, if both elements are involved. Unexpectedly, only dimer, trimer and tetramers of hDAT were identified after chemical cross-linking using Cu^2+^ or Cu^2+^-phenanthroline as oxidizing agent. Interestingly, the C243 cross-link was formed only to a limited extent, in contrast to the C306 cross-link. These data on human DAT are fully consistent with our results: We found a symmetric cluster of conformations, in which two C306 residues were pre-disposed for cross-link formation, but we were not able to observe such clusters with residue C243 in the interface. Notably, chemical cross-link formation is not necessarily representative of a conformational equilibrium situation, because it reports on reactivity, rather than on equilibrium distribution. Thus, a dimer has to exist only for a time period that is long enough for the chemical reaction to take place. hDAT is not chemically cross-linked at the plasma membrane under normal conditions, while under oxidizing conditions [13,14] the cross-linked dimers were a minor component. This is important because we can infer that the C243 dimer should be very rare, while the C306 dimer should be of low abundance, albeit higher than the C243-C306 heterodimer species. Such low frequency is precisely what we observed in our dataset.

TMH11 and TMH12 have been observed to be within a dimer interface in hSERT [17]. The similarity of hSERT and hDAT in many functional and biophysical aspects suggests that such interfaces may exist in hDAT as well. We identified TMH11 in the interface of heterodimers, whereas the involvement of TMH12 appears to be more complex. Of all the transmembrane helices among the SLC6 transporter family, only the conformation of TMH12 is not conserved between the bacterial homolog LeuT and human transporters [7–9]. This is attributable to a prominent kink in the center of the membrane. The dimeric LeuT crystal structure showed TMH9/12 at its interface. However, the kink in TMH12 makes the same dimer geometry virtually impossible. We still find TMH12 in the dimer interface, nonetheless the interface is limited to residues located predominantly towards the extracellular and possibly intracellular ends.

Sequence analyses carried out before solving the first LeuT structure uncovered a putative leucine heptad repeat in TMH2, which was suggested to be involved in transporter oligomerization of rGAT1 [44] and hDAT [21]. Surface targeting of both transporters was largely reduced and FRET analysis established a loss of oligomer formation in rGAT1 following mutagenesis of the respective leucine side chains to alanine. However, the crystal structures of LeuT, dDAT and hSERT did not confirm the existence of the predicted leucine heptad repeat [45]. A π-helix element in the middle of TMH2 creates a frame shift in the positioning of the helix residues with respect to a canonical α-helix. Hence, the leucine residues can no longer be aligned to form a heptad repeat. Furthermore, two of the four residues are not surface-exposed and cannot be involved in protein-protein interactions.

The C-terminal helix, directly following TMH12, is absolutely essential for the surface expression of hDAT and the related transporters [32,42,46,47], since it plays a key role in protein folding and trafficking, as well as in the interaction with the transporter core [48,49]. Moreover, a motif located in the C-terminal region is necessary for recognition by the SEC24C vs. SEC24D components of the ER export (COPII) machinery [47,50]. In addition, the C-terminus harbors the FREK sequence (residues 587–590), which is the binding site for the small ras-like GTPase Rin1, that is involved in PKC-mediated endocystosis of the transporter [51,52]. Cluster D revealed dimers which interacted only through the C-terminal helices, stabilized by a salt bridge and aromatic interactions between the protomers, but devoid of contacts with the transmembrane region. The C-terminal helix was embedded in the lipid headgroup region of the membrane, thereby affecting the lipid bilayer. This is indicative of a general biophysical property, which can possibly be adopted by every member of the SLC6 transporter family, and is also consistent with the specificity of SEC24 isoforms required for ER export of individual proteins. Namely, there are four human SEC24 isoforms (SEC24A-D) that recognize and bind some ~6000 membrane proteins encoded for in the human genome.

The entire ensemble revealed that we obtained a set of structures that clustered into 8 dimer conformations, consisting of four symmetric and four asymmetric dimers, showing in total 6 partially overlapping and consequently mutually-exclusive interfaces. Mapping the entire ensemble revealed that the putative interfaces covered a large part of the hDAT surface: it consisted of the scaffold domain comprising TMH 4, 9, but also TMH 3, 8, and 12 at their extracellular or intracellular ends. In addition, TMH 5 and 11, which are diagonally oriented at the transporter surface, extensively contribute to the dimer interfaces. It is remarkable that the four helices of the bundle domain (TMH 1, 2, 6 and 7) appear to be absent from the dimer interface, with the exception of the first turn of TMH6 until residue W311, which formed contacts in some dimers. Analysis of all trajectories revealed that on rare occasions (a total of 7 trajectories), transient dimers formed with the bundle domain as the core of the dimer interface. However, they were always unstable and separated within 0.5 μs. The lack of such dimers was not due to limited sampling, because from our ensemble of 512 independent simulations, we would have expected 20–40 dimers forming through random encounters. Importantly, the PMF profiles of these seven dimers were flat (Fig 6I), verifying the lack of dimer-stabilizing interactions. Collectively, these data suggest that hDAT establishes a force that opposes dimer formation at its bundle domain surface. Our data indicate that the bias is, at least, two-fold: i) hDAT seems to perturb the membrane to make encounters less likely. ii) Interactions of residues in the bundle domain with a second hDAT transporter are generally weak, because EL3 forms a rim-like structure at the transporter-lipid interface and is thereby floating in the membrane headgroup region, strongly limiting the extent of the transporter surface in direct contact with the dimer interface.

Biological membranes are a complex mixture of many types of lipids, asymmetrically and non-homogeneously distributed over the two leaflets. A full description of this complexity would be desirable, but is not yet achievable. The cholesterol content in the ER membrane is below 5%, and it is devoid of PIP2. Oligomerization of the homolog hSERT was shown to be indistinguishable between the ER membrane and a PIP2 free plasma membrane, suggesting that transporter oligomerization is largely insensitive to the membrane composition. The use of a POPC lipid membrane is therefore a good compromise, as it consists of the dominant phospholipid species in the plasma membrane as well as the ER membrane, though details might differ.

Not all membrane properties have the same importance for oligomerization. Experimental data showed that unexpectedly the cholesterol content does not play a major role [27]. Lipids form annular structures around proteins. The analysis of lipid order [53] revealed that the first layers of lipids showed increased ordering and therefore changed dynamics that differed from unperturbed lipids. The ordering of lipids does not differ much between the clusters A-H (S9 Fig) and the transient dimers which include the bundle domain in the interface, suggesting that the annular lipids do not play a major role in preventing dimer formation at the bundle domain. The hydrophobic region of membrane proteins does not always perfectly match with the thickness of the membrane. The hDAT is no exception as it shows regions which induce membrane thinning, while other parts of the hDAT lead to a thickening of the membrane (S10 Fig). The analysis of membrane thickness [53] indicated a pattern. The unstable transient dimers that included the bundle domain frequently showed areas of strong membrane deformation with opposing sign in close proximity originating from dimerization. In contrast, strong changes in membrane thickness across the interface were largely absent in the stable dimer, suggesting that mismatching membrane thickness might play a role in preventing dimer formation at the bundle domain by inducing strong membrane deformations.

**Fig 8:**
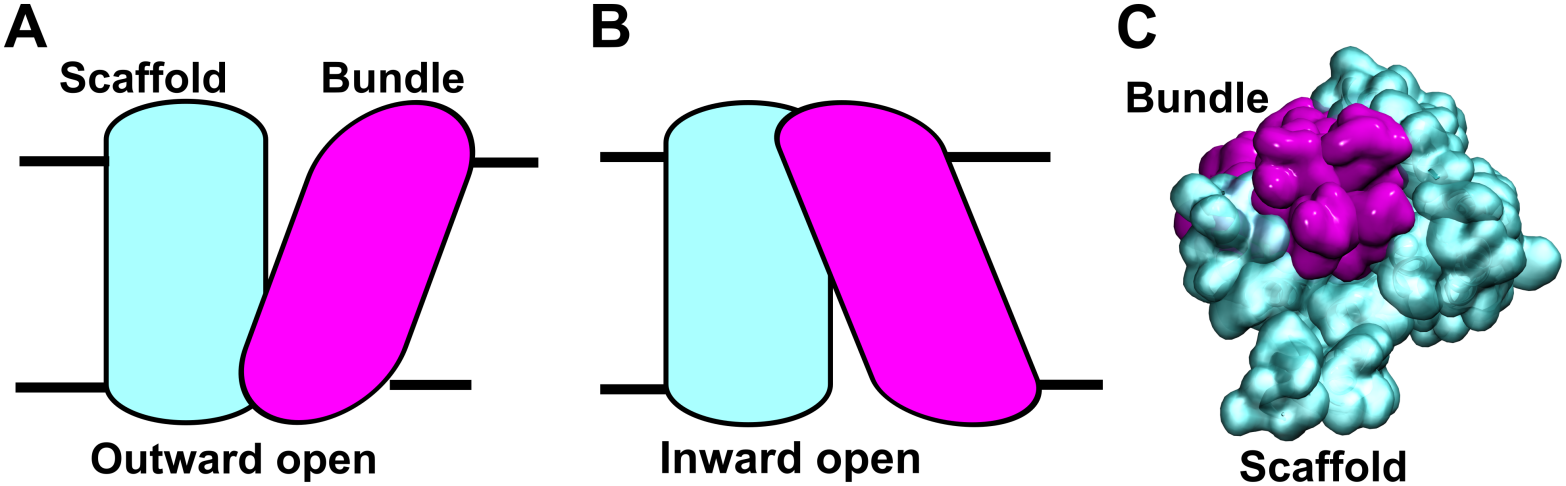
Conformational changes during transport. The schematic representation shows hDAT A) in the outward-open and B) in the inward-open conformation, exemplifying the changes in protein geometry during transport. C) Visualization from the intracellular side of the scaffold (cyan) and bundle domains (magenta) on a surface representation of the outward-facing conformation. The orientation in panel C is identical to the orientation of reference protomer in Figs 3 and 4.

Our studies of LeuT and hDAT simulations [54,55] showed that the scaffold domain anchors and stabilizes the transporter in the membrane. In addition, crystal structures showed that the bundle domain, which moves during the transport cycle (Fig 8), comprises only a small fraction of the membrane-exposed surface of hDAT. The work required for pushing against the membrane to allow for bundle domain movements is therefore limited. Any larger interaction surface including TMH 1, 2, 6 or 7 would block transport by locking the bundle domain in one single conformation, thereby impeding the switch between the inward- and outward-facing conformations, a movement necessary for the transport cycle (Fig 8A and 8B). We also identified residue C306 within the interface; this residue is located in EL3, and in close proximity to TMH6 (as shown in Fig 5C). However, the frequency of formation of this particular dimer is relatively low. The dimer was stabilized by a double inter-molecular salt bridge between R304 and E307, though the same residues could also form an intra-molecular salt bridge, thereby weakening the interaction across the dimer interface. Mutation of either of the two charged residues (R304 and E307) resulted in a strong reduction of cross-linked hDAT, following oxidative treatment [30]. This distinct symmetric dimer interface, including EL3, could still allow for transport, since the interacting side chains of R304 and E307 are long and flexible and can permit a large range of adjustments whilst sliding relative to one another. The direction of conformational changes during the transport cycle is such that this last segment of EL3 would slide in parallel, and thus limit the impact of this dimer geometry on the conformational changes in the transport cycle. The R304A and E307A mutants showed reduced expression but a gain in transporter function [30], providing further evidence in support of our data. The salt bridge may be able to switch between the inter- and intra-molecular arrangements. This in turn allows for conformational changes in the transport cycle, which may not be necessary for the R304A and E307A mutants.

As discussed above, several oligomeric contact points identified in this study have already been observed experimentally. Our results suggest the existence of a range of symmetric and asymmetric DAT dimers rather than one well-defined oligomeric structure. The coarse-grained force field, which is necessary to reach the time scales required in this study, comes with the limitation that approximations in the short-range interactions needed to be introduced in the description of the system. Application of the DAFT approach to study GPCRs dimerization showed reproduced experimental data [31,56–58], indicating that the coarse-grained system does nevertheless allow for direct study of protein dimerization. It was recently indicated that protein-protein interactions might be too strong [59] in the Martini Force Field. This leads to an overstabilization of dimers, which most affects the weak and transient unstable dimers. The hDAT dimer geometries observed in the 8 clusters are least affected, because the timescale for dimer separation is on the timescale of minutes, as experimentally measured for hSERT [26–28]. Overstabilization would therefore not significantly affect the geometry of dimer, but the PMF profiles might be exceedingly attractive. It will be important to experimentally confirm the predicted dimer interfaces and establish whether all interfaces would equally contribute to larger oligomer structures. The observation of a set of possible hDAT-hDAT interface and oligomeric structures has additional functional implications that warrants further experiments. It will be rather unlikely that one defined positive or negative allosteric interaction would exist between protomers. In this light, it is surprising that a very recent study [25] reported that a DAT dimer likely consists of one active and one non-active protomer as has been suggested for SERT [43]. Such a binary impact (active or inactive) is difficult to reconcile with the range of oligomers and interfaces as reported in this study or observed for SERT in single molecule studies [28].

The lipid species PIP2 is well known for its interaction with—and regulation of—membrane proteins. PIP2 is a negatively-charged phospholipid that resides in the intracellular membrane leaflet of the plasma membrane and accounts for 1% of the total membrane lipid content. We have previously reported that: (i) PIP2 interacts with monoamine transporters, (ii) the size and stability of transporter oligomers depends on PIP2 [28] and (iii) that transporter efflux is PIP2-dependent as well [37], thereby affecting behavioral response to psychostimulants [38]. The hSERT residues SERT-K352 and SERT-K460 are exposed on the cytosolic membrane and were found to play a role in the PIP2-mediated effects [37]. Here we analyzed the electrostatic field generated by hDAT dimers. A subset of dimer conformations showed a positive electrostatic field that connected the two protomers, whilst bridging over the lipid bilayer. It is conceivable that these extended areas of positive electrostatic fields could act as the regions that attract PIP2 lipids, since PIP2 carries a total charge of -5. The size of the PIP2 headgroup would than allow for interactions with both hDAT protomers. These areas included three regions on hDAT: a) IL1 and IL4, b) IL5 and c) IL2, TMH1 and IL3. The loops carry a large number of positively-charged lysine and arginine residues, including the sites, which were found to interact with PIP2 in the homolog SERT (SERT-K352 in IL3 and SERT-K460 in IL4). Removal of these charges would reduce the positive field, hence reducing the interaction with PIP2. It is worth mentioning that three of these clusters were asymmetric; the exception being the sparsely populated and symmetric cluster C. In hSERT, depletion of PIP2 led to a dramatic reduction of higher oligomers, while the dimer population remained mostly unchanged. Assuming that hSERT and hDAT behave comparably, our results indicate that the highly populated symmetric dimers would not be affected by PIP2 binding, while the highly populated asymmetric dimers would be. We can therefore surmise that the assembly of oligomeric structures larger than dimers ought to involve asymmetric interfaces that are PIP2-sensitive.

## Conclusion

The oligomeric arrangement of the monoamine transporters differs from ion channels and ionotropic receptor in that the latter form well-defined trimeric, tetrameric or pentameric structures, while the former show an oligomeric size distribution that decreases mono-exponentially, indicating dynamic exchange, thereby excluding the possibility of a single well-defined oligomeric arrangement. Structures larger than dimers require at least two non-overlapping interfaces. Our simulations elucidate that hDAT forms stable dimers through 6 different interfaces, including the already identified interfaces comprised of residues C243 and C306, TMH11 and TMH12. Importantly, the bundle domain appears to be excluded from these interfaces, thus allowing for efficient motions during the transport cycle. The number of transporters must be high to support fast neurotransmitter clearance, as neurotransmitters need to be removed faster than they are released to prevent large concentration buildup and systemic spillover. The observed interface distributions support a high transporter density via several interfaces, while maintaining maximum transporter function, by avoiding transport impeding interactions at the bundle domain, that must be free to move during the transport cycle.

## Materials and methods

### Human DAT model creation

Homology models of the human dopamine transporter (hDAT) were created from residues 44 to 602 based upon the outward-open crystal structure of the *Drosophila melanogaster* dopamine transporter (dDAT) [7] (PDB ID: 4XP1) using modeller 9.15 [60] which share 72% sequence identity of their transmembrane region (TMH1 to TMH12). The sequence alignment is given in the S11 Fig. The models contained 2 Na^+^ and 1 Cl^-^ ions in their respective binding sites, and a single dopamine molecule bound in the central binding site as observed in the crystal structure of dDAT. A previously modeled extracellular loop 2 (EL2) [54] was introduced by fitting to the structurally shared connecting secondary structure elements of helix TMH3 and the helix of EL2, because the EL2 of dDAT was truncated for crystallization and showed extensive crystal contacts with the co-crystallized antibody. The 250 models, produced by applying the automodel procedure using the refinement protocol “normal”, were scored and sorted by their discrete optimized protein energy (DOPE) score [61]. The best 20 of these models were re-ranked by their root mean-squared deviation (RMSD) of the Cα atoms from the template. The model with the lowest RMSD has a DOPE score of -78938, and shows a Ramachandran plot (S12 Fig), where 94.0% of residues are in the most favourable region, 5.2% in the additional allowed region, 0.4% in the generously allowed region and 0.4% in the disallowed region. The quality of the model was also assessed using the QMEAN score [62], which shows that the local quality of the model is especially high for the transmembrane region (S13 Fig). This model was inserted as apo protein into the DAFT work flow. The Cl^-^ and two Na^+^ ions need to be removed from the structure, because these ions cannot be described correctly by the Martini coarse-grained force field. Ions can only be represented by including their first hydration shell as one single particle, while these three ion are stripped of their hydration shell when bound to hDAT. We therefore also removed dopamine, because its binding depends on the present of bound sodium.

### DAFT-workflow

The DAFT (Docking Assay For Transmembrane components) approach [31] allows to identify protein-protein interactions and binding orientations. It uses molecular dynamics simulations for exploring the conformational search space, therefore explicitly including the entropic component associated with the dimerization event. Moreover, it uses a coarse grain representation to allow for a microsecond to millisecond time scale. The DAFT workflow consists of several different modules coupled together. Conversion of the all atom fine-grained (FG) homology model of apo hDAT to the coarse-grained (CG) representation of the ElNeDyn CG implementation of the Martini force field was done by the martinize module [63]. Then, 512 hDAT dimer systems were created with random relative orientations in the membrane plane and a center of mass distance between the two protein molecules of 8.4 nm, which resulted in a typical minimal distance between protomer of > 2.5 nm. The only exception was the orientation in which the C-terminal helices of both protomers face each other, in which case the minimal distance was close to 2.0 nm. The time evolution of trajectories starting from these conformation showed that the closer distance was without bias towards a specific dimer formation during the DAFT simulations as simulations drifted away from this initial orientation. The membrane was built around the proteins using POPC lipids by the insane module [64], adding CG water and ions (Fig 1). Each system contained 172 to 190 lipids per leaflet, the salt (NaCl) concentration was set to 150 mM. The systems were first energy minimized, than equilibrated with NVT ensemble simulations while restraining the protein. Production runs (2.0 μs each) with NPT ensemble were carried out using a timestep of 20 fs, the v–rescale thermostat [65] was used to maintain the temperature at 310 K, the weak coupling barostat applied to keep the pressure at 1 bar [66]. The electrostatic interactions were defined according to the Martini force field by a coulomb type shift and the values are switched between 0 to 1.2. The Van der Waals interactions were represented by Lennard-Jones potentials using the shift type to 0 between 0.9 to 1.2 nm.

### Fine-grained simulations

Selected final structures of the CG systems were back-mapped into FG all atom representation using the backward module [40] using two steps of energy minimization and four steps of system relaxation. The resultant structures were simulated using the amber99sb-ildn force field [67] for the transporter and applying the Berger parameters for the lipids [68], as this combination of force fields showed the best performance [69]. For system relaxation, the protein was restrained by applying 1000, 100, 10 and 1 kJ·mol^-1^ restraints on the protein, each for one nanosecond. Further, the production run was carried out for 100 ns. The temperature was maintained at 310 K using the v-rescale temperature coupling [65] and the pressure was maintained semi-isotropically at 1 bar using the weak coupling barostat [66]. The pressure coupling time constant was set to 1 ps and the compressibility to 4.5×10^−5^ bar^-1^. Long range electrostatics interactions were represented by the particle mesh Ewald method [70] with a cutoff of 1.0 nm. The Van der Waals interactions were imposed using Lennard Jones potential using the 1.0 nm cutoff. All the bonds are constrained using LINCS [71].

### SMD and PMF calculations

To quantify the energies involved in dimer stabilization we carried out PMF calculations using the CG system representation. We selected two dimers from each cluster and also included all seven system of the DAFT dataset which showed dimer contacts that included the bundle domain at any timepoint of the 2.0 μs long trajectories. The center of mass distance was used as the reaction coordinate. The starting conformations for the PMF calculations were develop by a series of steered MD (SMD) simulations [72], in which we increased the center of mass distance between the protomers. The velocity for the movement of the reference point was set to 0.025 nm/ns, which is in the range of the fastest diffusion as measured by protein diffusion in the unbiased simulations of individual protomers before dimerisation. Large membrane deformation by a pulling velocity, which is much faster than normal diffusion can thereby be avoided. To allow for sufficient protomer separation, we extend the membrane in the direction of the reaction coordinate by 5 nm and filled the additional space with lipids, water and ions. While restraining the hDAT protomers, these extended system were equilibrated using first a time step of 2 fs for 10 ns to relax initial structural strain, followed by a 100 ns long equilibration using a 20 fs time step.

During the SMD simulations we restrained the relative orientation of the protomers using the enforced rotation module [73] implemented in Gromacs [74] to limit the conformational search space, thereby making sampling of the reaction coordinate affordable. This procedure is formally correct and allows to set the bound and the unbound state into an energetic relationship. The restraints allow to circumvent the sampling problem at intermediate distances by limiting the exploration of phase space to the reaction coordinate. Quantification of the complete free energy hypersurface would require extensive sampling of the hDAT binding and unbinding events, for which the single molecule data of the hSERT indicate that the process requires minutes to equilibrate [28].

From these SMD trajectories we extracted hDAT dimer conformations along the reaction coordinate from the first 1.6 nm at 0.1 nm intervals. Each structure was used as the starting point for an umbrella simulation of 100 ns, applying the enforced rotation module to maintain relative hDAT orientation. Moreover a harmonic potential of 1000 kJ/mol/nm was applied between the center of mass of two protomers, acting on their backbone. Additional umbrella windows with a 0.025 nm spacing and a restraining force of 5000 kJ/mol/nm were added within the first 0.4 nm to enhance sampling at shorter distances, resulting in a total of 32 umbrellas per PMF profile. Block analysis showed that all systems relaxed within 50 ns; we therefore use the second half of each umbrella trajectories for the PMF analysis using WHAM [75] and applying the bootstrap method [76].

## Supporting information

S1 Fig Convergence of the DAFT ensemble. The energy of non-bonded interactions (vdW and electrostatics) between the dimers in the whole ensemble was plotted vs time. The distribution of energy values were represented in the form of vigintiles (5% quantiles), which splits the data into 21 levels. These vigintiles have a spectral color scheme: the minimum value is colored in pink, followed by a rainbow to reach the central vigintile colored in red. The second half of vigintiles is colored with inverted colors scale from red to pink. In addition, the mean value of all energies is shown as black line. The plot levels off towards the end and the mean value and the central vigintile overlap, indicating at robust but not completed convergence. During 2.0 μs of simulation time, the monomers diffuse and over 60% interacted at the end of the simulations. Although convergence is not complete within the 2.0 μs, it is robust enough to allow for analysis of the conformations and interacting residues in the hDAT dimer interface.
(TIF)

S2 Fig Overlay of final structures. All final 512 dimer structures of the DAFT simulations are overlayed. These are all fitted to protomer A and shown in semitransparent representation so that each structure alone appears in faint grey. The overlay of the 512 protomer A structures accumulates intensity and results in the black structure in the centre. In contrast, protomer B is non-homogeneously distributed and oriented relative to protomer A. The overlay of these 512 protomer B structures (also semitransparent) leads to the circular ring-like shape surround protomer A. The relative orientations with high numbers (consistent of the location of the main 8 clusters) lead to darker grey regions, while relative orientation with low probability are less dark. The arrow marker in the left lower corner indicates the membrane plain (green/red) and the membrane normal in blue.
(TIF)

S3 Fig Stability of dimers in fine-grained all atom simulation. A representative back-mapped configurations for every cluster was simulated for 100 ns. RMSD relative to the starting structure of hDAT dimers is shown as a function of time. The largest contribution to the RMSD value came from small wiggling motion of the two protomers relative to each other. The interaction within the dimer interfaces remained stable, also the overall geometry.
(TIF)

S4 Fig Residue involved in the dimer interface. Number of interactions of residues closer than 0.5 nm observed in the final 100 ns (5 frames) summed over all simulations associated with one cluster. Transmembrane helix residues are highlighted at the bottom. Insets show the interacting residues across the dimer interface. For the sake of clarity only interactions occurring more than five times are considered. The line thickness is related to the relative frequency of the interaction. Circles in the insert indicate an interaction between identical residue across the dimer interface.
(TIF)

S5 Fig Starting structures. Backbone representations are shown for the starting structures of every SMD/PMF calculation from cluster A-H, viewed from the membrane plane and from the extracellular site.
(TIF)

S6 Fig Starting structures. Backbone representations are shown for the starting structures of every SMD/PMF calculation of dimers, which showed the bundle domain in the dimer interface, viewed from the membrane plane and from the extracellular site. These correspond to starting structures for the simulations of Fig 6I.
(TIF)

S7 Fig Deviation from reference position normal to the reaction coordinate PMF profile. A-I) Deviation from the reference position normal to the reaction coordinate of protomer separation. The deviation is shown for every umbrella window of the PMF profiles, as shown in Fig 6. The averages are taken over the second half of each trajectory. The color code is consistent with Fig 6.
(TIF)

S8 Fig Rotation from reference orientation of the PMF profile. A-I) The deviation from the reference orientation is shown for every umbrella window of the PMF profiles, as shown in Fig 6. Averages are taken over the second half of each trajectory. The color code is consistent with Fig 6. The standard deviation of the angel values remains below 0.075° for all systems.
(TIF)

S9 Fig Membrane order parameter. A-I) Membrane order parameter is shown for two representative systems per cluster. These are the same systems as used in the PMF calculations, labeled according to Fig 6 (cyan and magenta color of panel label). We use the trajectories of the first umbrella window and averaged the membrane order parameter over the second half of each trajectory. The starting structures of each simulation are the respective final structures of the DAFT simulations. The systems are shown from the intracellular side. The ordering of lipids is estimated using a second-rank order parameter defined as: S = 1/2 * (3 * <cos^2^(θ)> − 1), where θ is the angle between the membrane normal and the bond between two successive beads of the Martini lipid model. The brakets < > represent ensemble averaging. A value of 1 indicates that the lipids would be perfectly aligned with the axis, while -0.5 indicates an orientation parallel to the membrane plane. A decrease/increase in ordering of the annular lipids is visible in the annular structure surround hDAT. The lipids directly attached to hDAT show the strongest deviation from bulk order parameter. Panel 1–7 shows the membrane order parameter of the transient dimers coloured according to Figs 3B and 6I. Panels 1–7 do not indicate a pattern of specific deviation that would set these systems apart from the members of cluster A-H. This indicates that lipid ordering is not a driving force that prevents dimerization at the bundle domain.
(TIF)

S10 Fig Membrane thickness. A-H) Membrane thickness is shown for two representative systems per cluster. These are the same systems as used in the PMF calculations, labelled according to Fig 6 (cyan and magenta color of panel label). We used the trajectories of the first umbrella window and averaged the membrane thickness over the second half of each trajectory. The starting structures of each simulation are the respective final structures of the DAFT simulations. The systems are shown from the intracellular side. Panel 1–7 show membrane thickness of the transient dimers coloured according to Figs 3B and 6I. Panels 1–7 show in most system a strong change of membrane thickness across the dimer interface, placing areas of high membrane thickness next to areas of low membrane thickness. Overall an area of increased membrane thickness at one protomer is paired with an area of low membrane thickness of the second protomer. The mismatch in pairing of membrane thickness might contribute to the inability to form dimers, which include the bundle domain. The same mismatch was not observed for the stable dimers from cluster A-H.
(TIF)

S11 Fig Sequence alignment. Sequence alignment of the dopamine transporters from human and *Drosophila melanogaster*. Residues are coloured according to the color code of clustal.
(TIF)

S12 Fig Ramachandran plot of the best hDAT model. The Ramachandran plot represents a quality assessment of the backbone geometry of the hDAT model: 94.0% of residues are in the most favourable region, 5.2% in the additional allowed region, 0.4% in the generously allowed region, and 0.4% are found the disallowed region of the Ramachandran plot.
(TIF)

S13 Fig Qualitative Model Energy Analysis (QMEAN) model quality analysis. QMEAN is a scoring function that allows for assessing model quality using structural descriptors including local geometry, structural compactness, secondary structure, and solvation. The QMEANBrane score is specifically optimized for transmembrane proteins. A) Comparison of QMEANBrane score to reference dataset of membrane proteins. B) Mapping of the local QMEANBrane score on the model of hDAT, C) Per residues score showing per residue model quality. D) Z-score.
(TIF)

S1 Table Cluster statistics. Summary of the number of dimer per cluster at 2μs as observed in Fig 2. Both off-diagonal clusters were merged in the asymmetric dimers.
(DOC)

S2 Table PMF profile integrals. The PMF profiles shown in Fig 6 are integrated and the integrals reported. The energy at full separation are set to zero. The color code of the cluster column is consistent with Fig 6.
(DOC)
