# Supplementary figures and images for: Dopamine transporter oligomerization involves the scaffold domain, but spares the bundle domain
Source: PLoS Comput Biol. 2018 Jun 6;14(6):e1006229. doi: 10.1371/journal.pcbi.1006229 (PMC6005636; doi:10.1371/journal.pcbi.1006229)

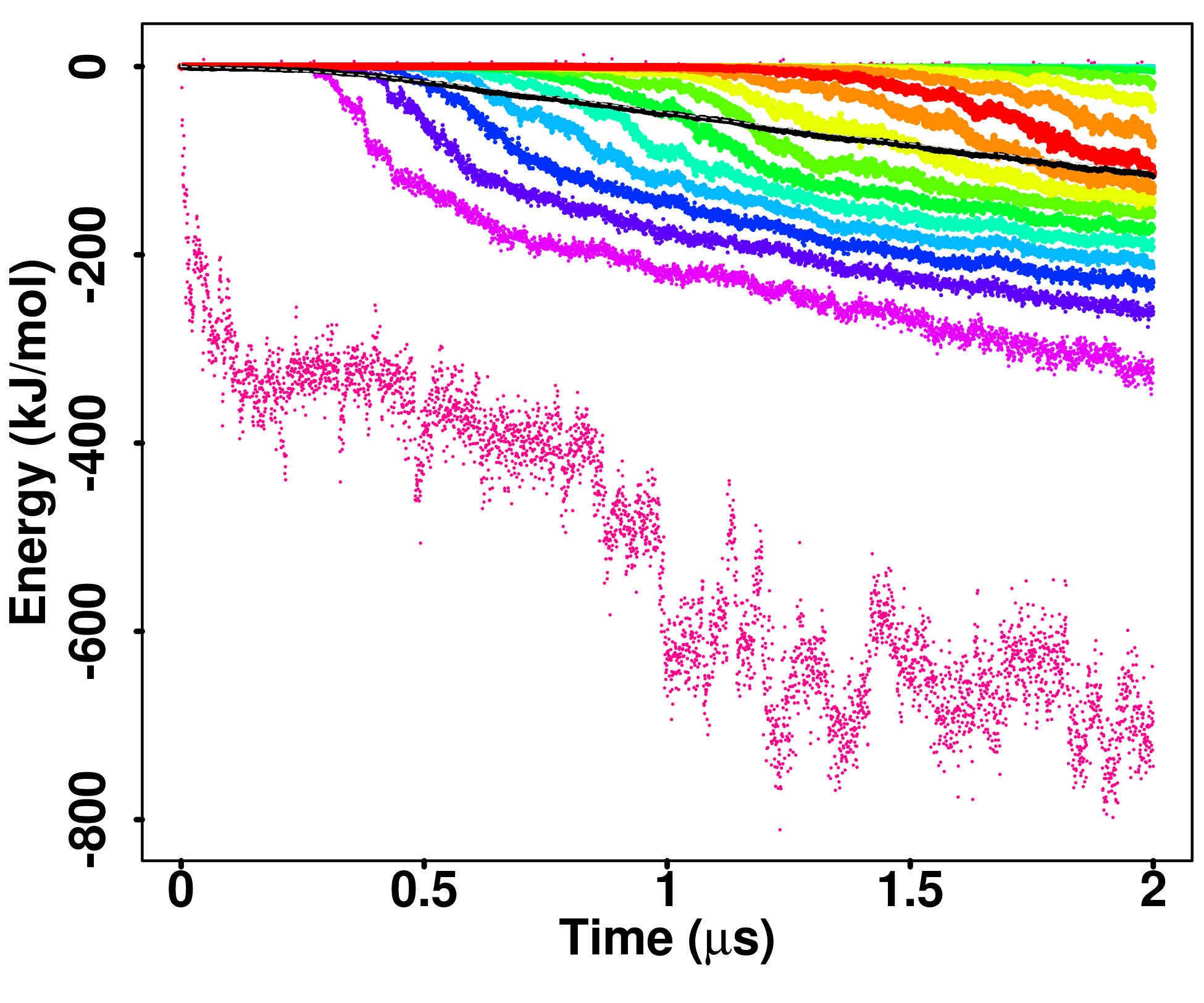

Supplement: S1 Fig — The energy of non-bonded interactions (vdW and electrostatics) between the dimers in the whole ensemble was plotted vs time. The distribution of energy values were represented in the form of vigintiles (5% quantiles), which splits the data into 21 levels. These vigintiles have a spectral color scheme: the minimum value is colored in pink, followed by a rainbow to reach the central vigintile colored in red. The second half of vigintiles is colored with inverted colors scale from red to pink. In addition, the mean value of all energies is shown as black line. The plot levels off towards the end and the mean value and the central vigintile overlap, indicating at robust but not completed convergence. During 2.0 μs of simulation time, the monomers diffuse and over 60% interacted at the end of the simulations. Although convergence is not complete within the 2.0 μs, it is robust enough to allow for analysis of the conformations and interacting residues in the hDAT dimer interface. (TIF) [file pcbi.1006229.s001.tif]

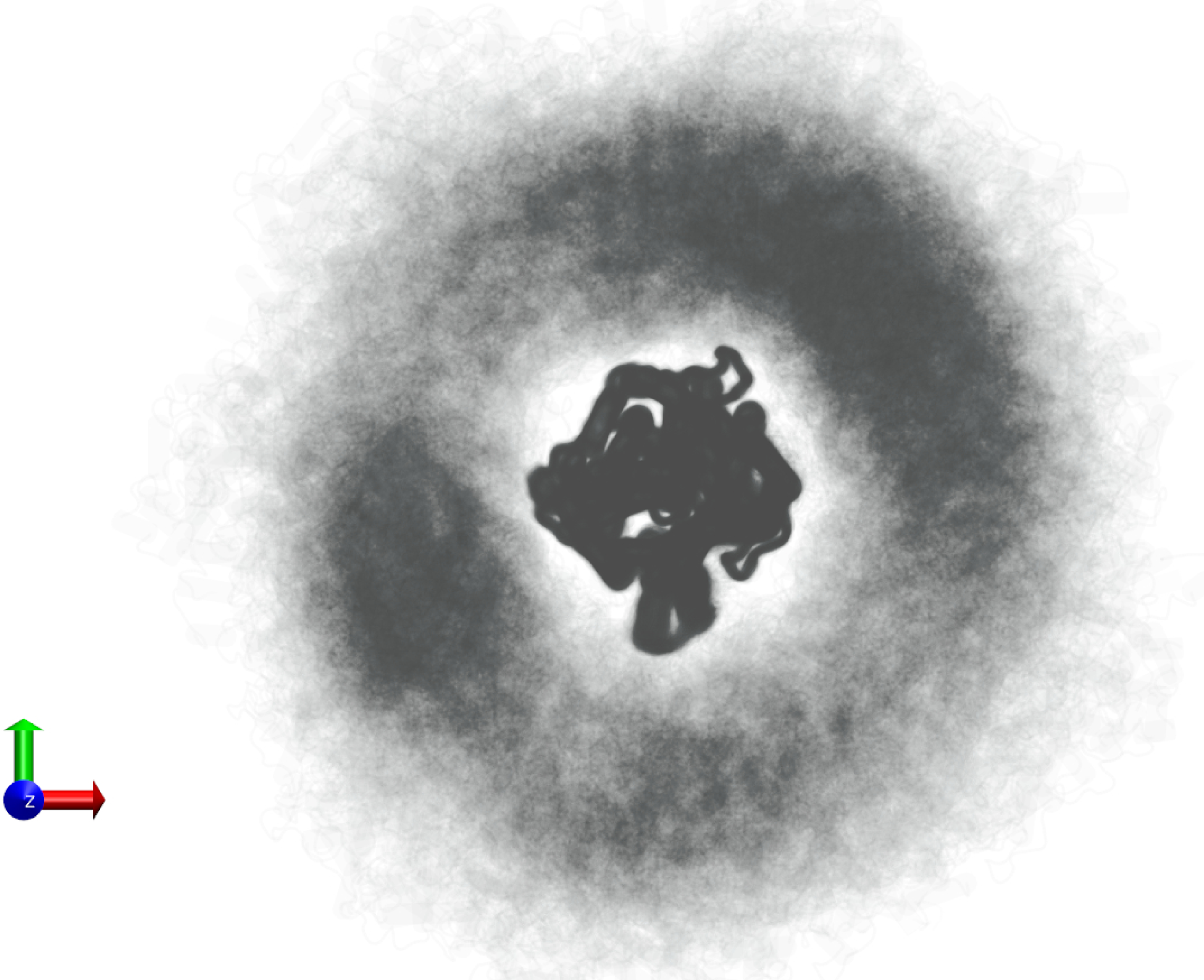

Supplement: S2 Fig — All final 512 dimer structures of the DAFT simulations are overlayed. These are all fitted to protomer A and shown in semitransparent representation so that each structure alone appears in faint grey. The overlay of the 512 protomer A structures accumulates intensity and results in the black structure in the centre. In contrast, protomer B is non-homogeneously distributed and oriented relative to protomer A. The overlay of these 512 protomer B structures (also semitransparent) leads to the circular ring-like shape surround protomer A. The relative orientations with high numbers (consistent of the location of the main 8 clusters) lead to darker grey regions, while relative orientation with low probability are less dark. The arrow marker in the left lower corner indicates the membrane plain (green/red) and the membrane normal in blue. (TIF) [file pcbi.1006229.s002.tif]

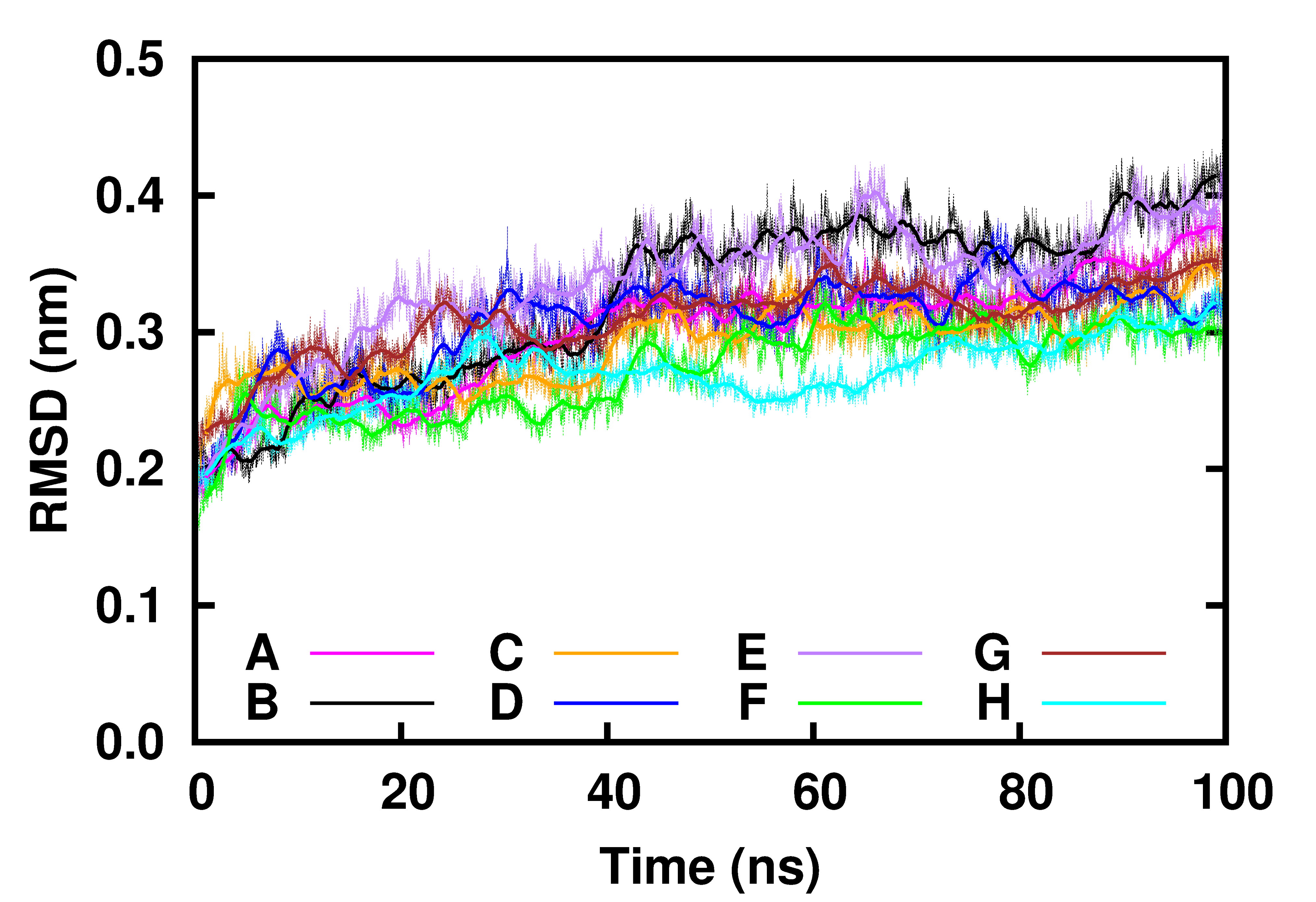

Supplement: S3 Fig — A representative back-mapped configurations for every cluster was simulated for 100 ns. RMSD relative to the starting structure of hDAT dimers is shown as a function of time. The largest contribution to the RMSD value came from small wiggling motion of the two protomers relative to each other. The interaction within the dimer interfaces remained stable, also the overall geometry. (TIF) [file pcbi.1006229.s003.tif]

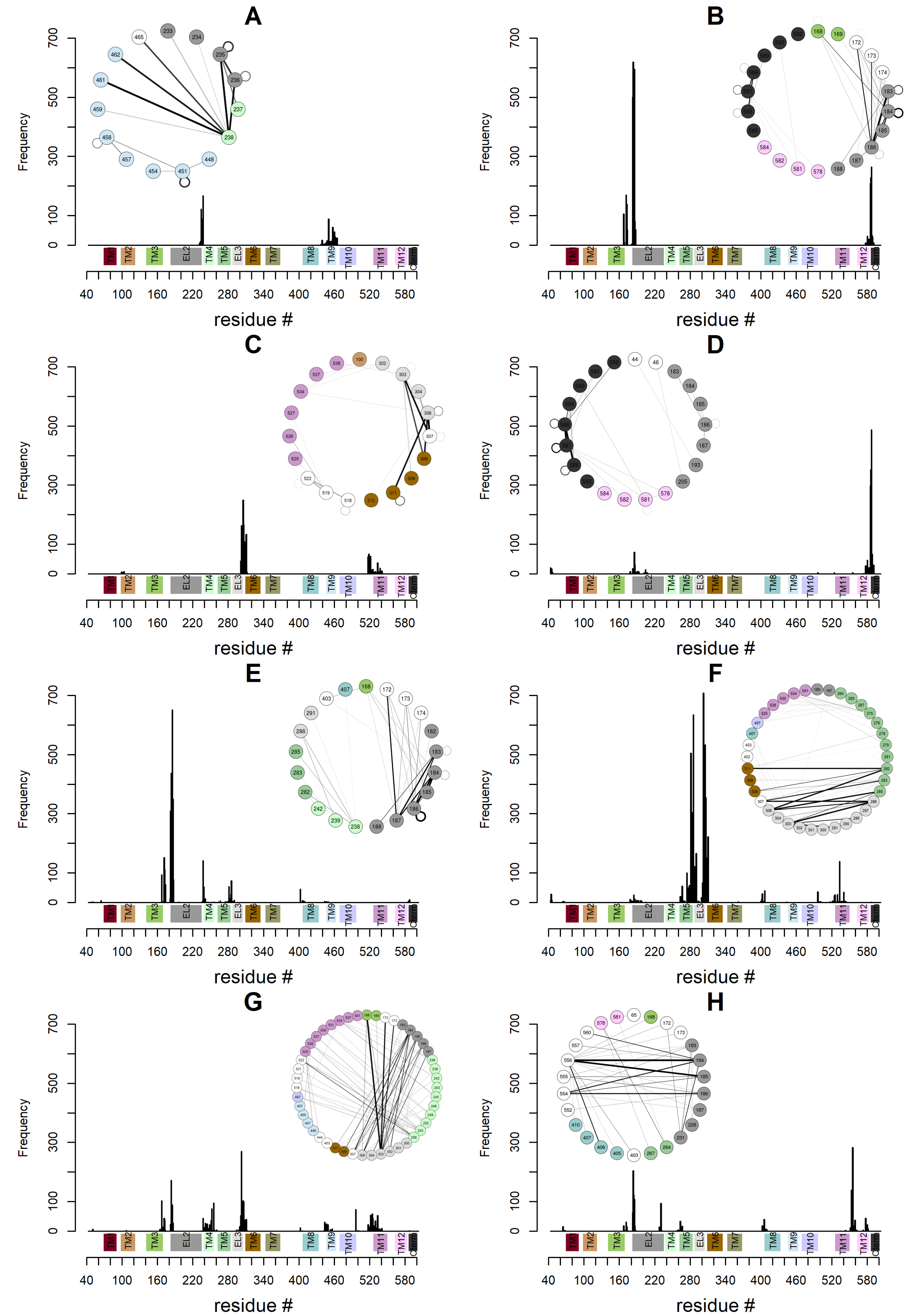

Supplement: S4 Fig — Number of interactions of residues closer than 0.5 nm observed in the final 100 ns (5 frames) summed over all simulations associated with one cluster. Transmembrane helix residues are highlighted at the bottom. Insets show the interacting residues across the dimer interface. For the sake of clarity only interactions occurring more than five times are considered. The line thickness is related to the relative frequency of the interaction. Circles in the insert indicate an interaction between identical residue across the dimer interface. (TIF) [file pcbi.1006229.s004.tif]

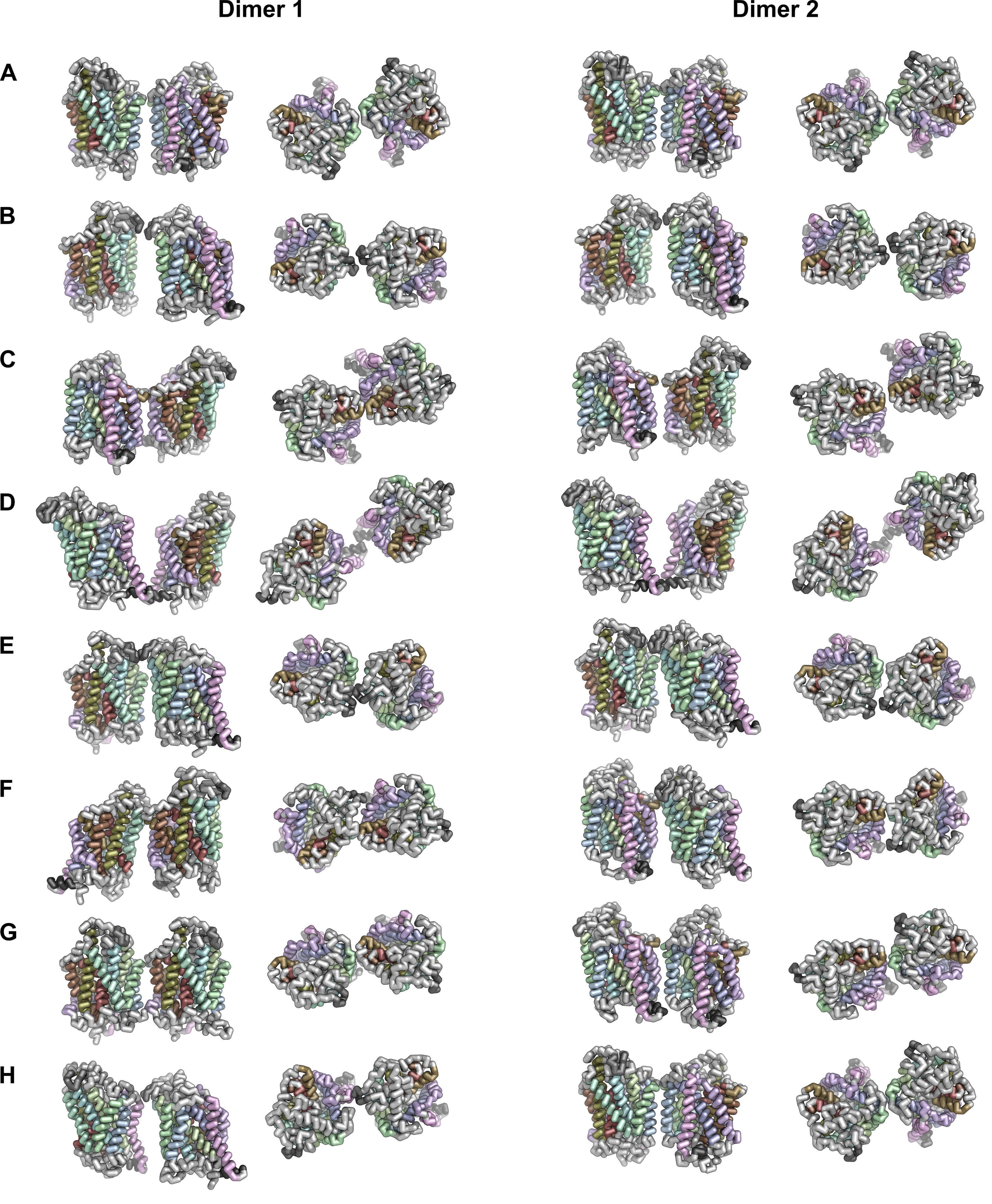

Supplement: S5 Fig — Backbone representations are shown for the starting structures of every SMD/PMF calculation from cluster A-H, viewed from the membrane plane and from the extracellular site. (TIF) [file pcbi.1006229.s005.tif]

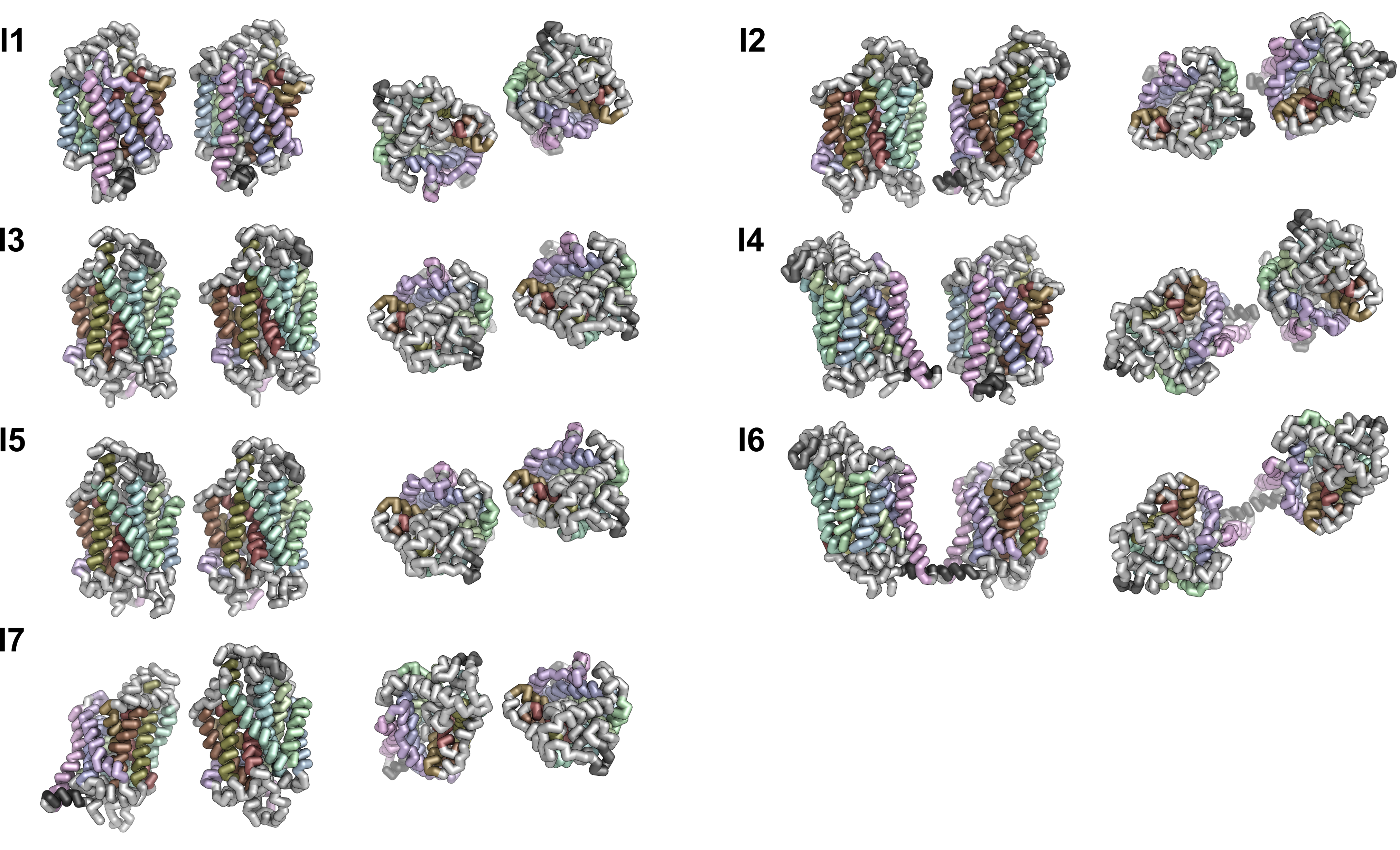

Supplement: S6 Fig — Backbone representations are shown for the starting structures of every SMD/PMF calculation of dimers, which showed the bundle domain in the dimer interface, viewed from the membrane plane and from the extracellular site. These correspond to starting structures for the simulations of Fig 6I. (TIF) [file pcbi.1006229.s006.tif]

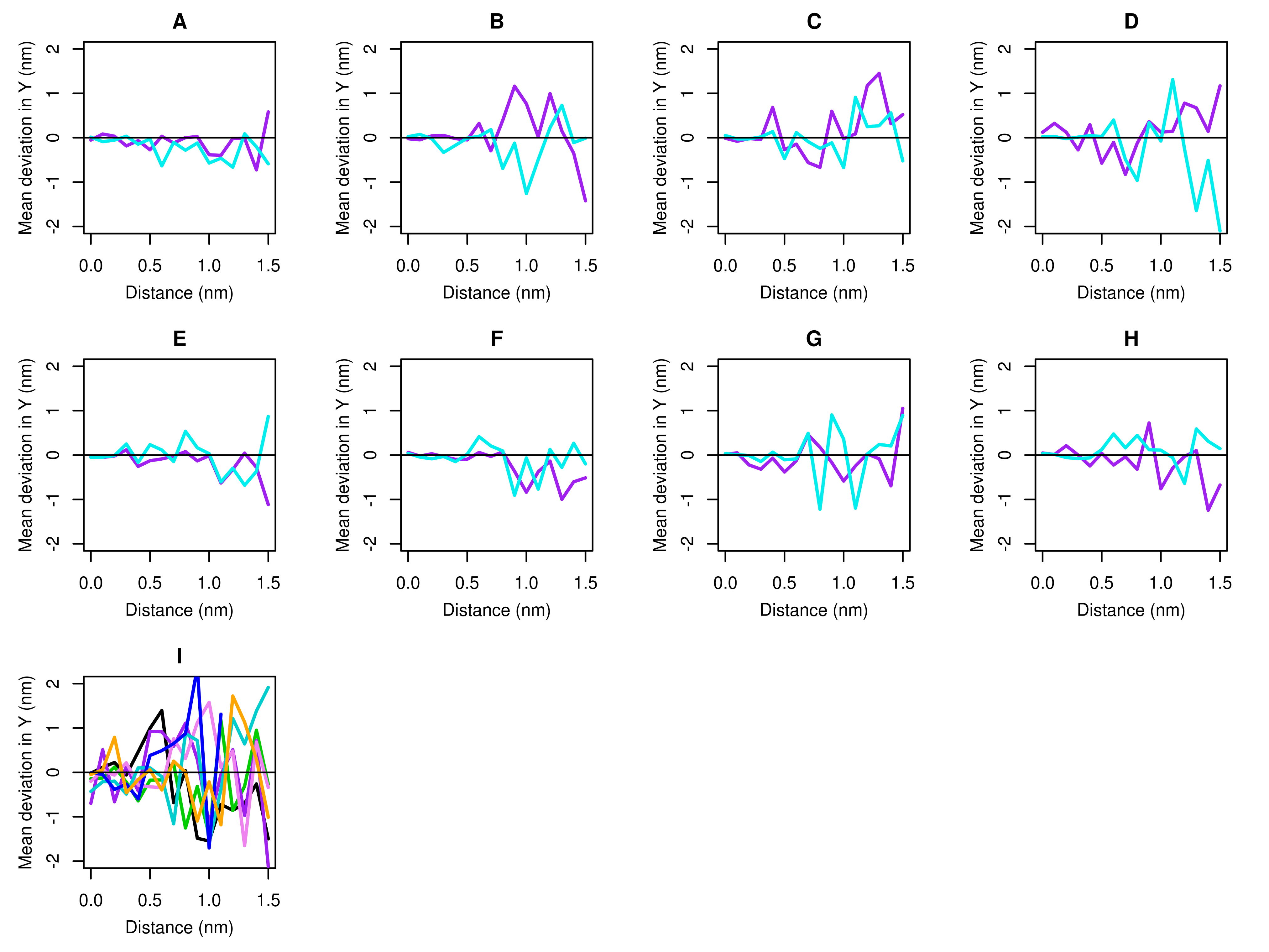

Supplement: S7 Fig — A-I) Deviation from the reference position normal to the reaction coordinate of protomer separation. The deviation is shown for every umbrella window of the PMF profiles, as shown in Fig 6. The averages are taken over the second half of each trajectory. The color code is consistent with Fig 6. (TIF) [file pcbi.1006229.s007.tif]

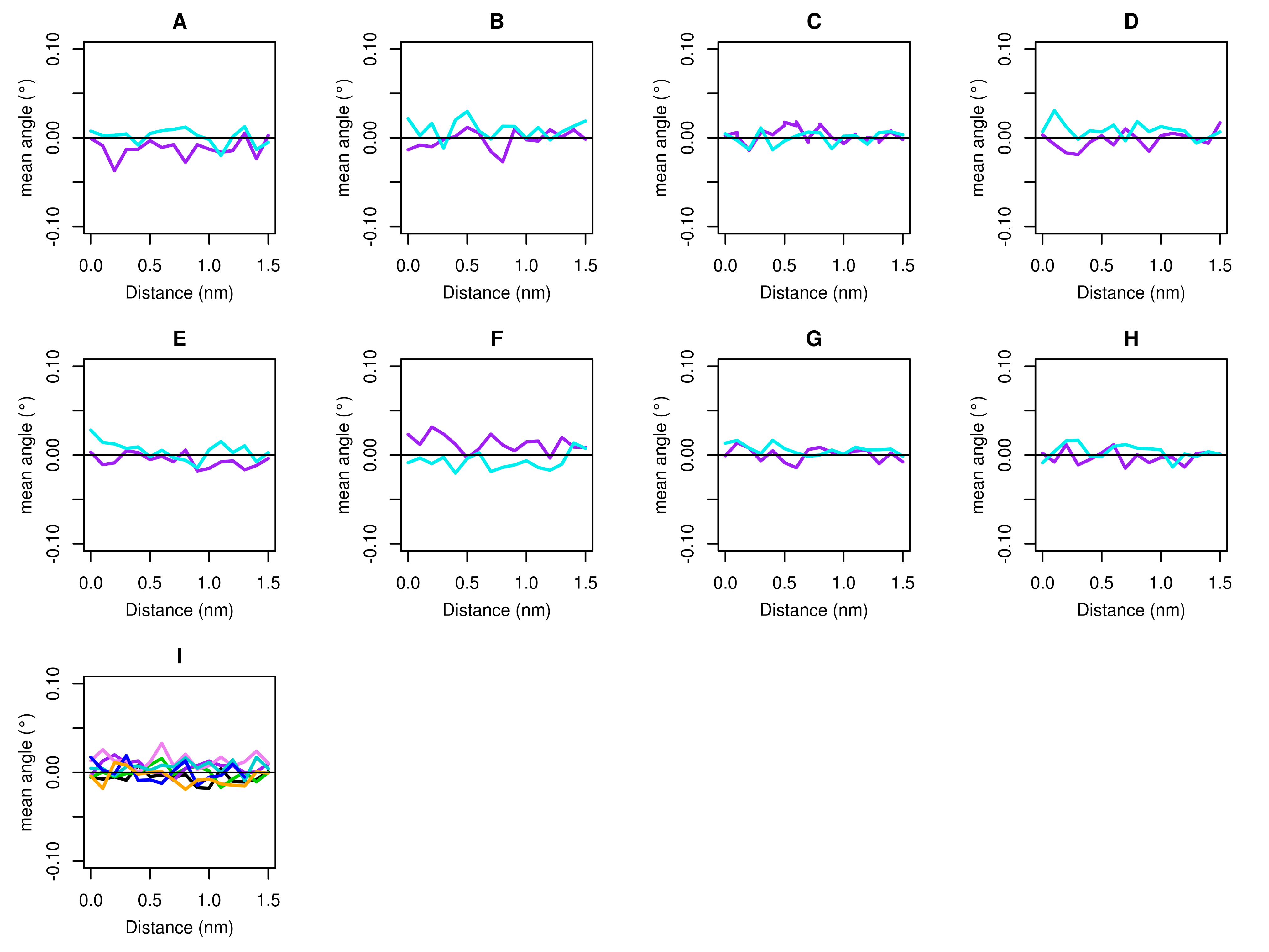

Supplement: S8 Fig — A-I) The deviation from the reference orientation is shown for every umbrella window of the PMF profiles, as shown in Fig 6. Averages are taken over the second half of each trajectory. The color code is consistent with Fig 6. The standard deviation of the angel values remains below 0.075° for all systems. (TIF) [file pcbi.1006229.s008.tif]

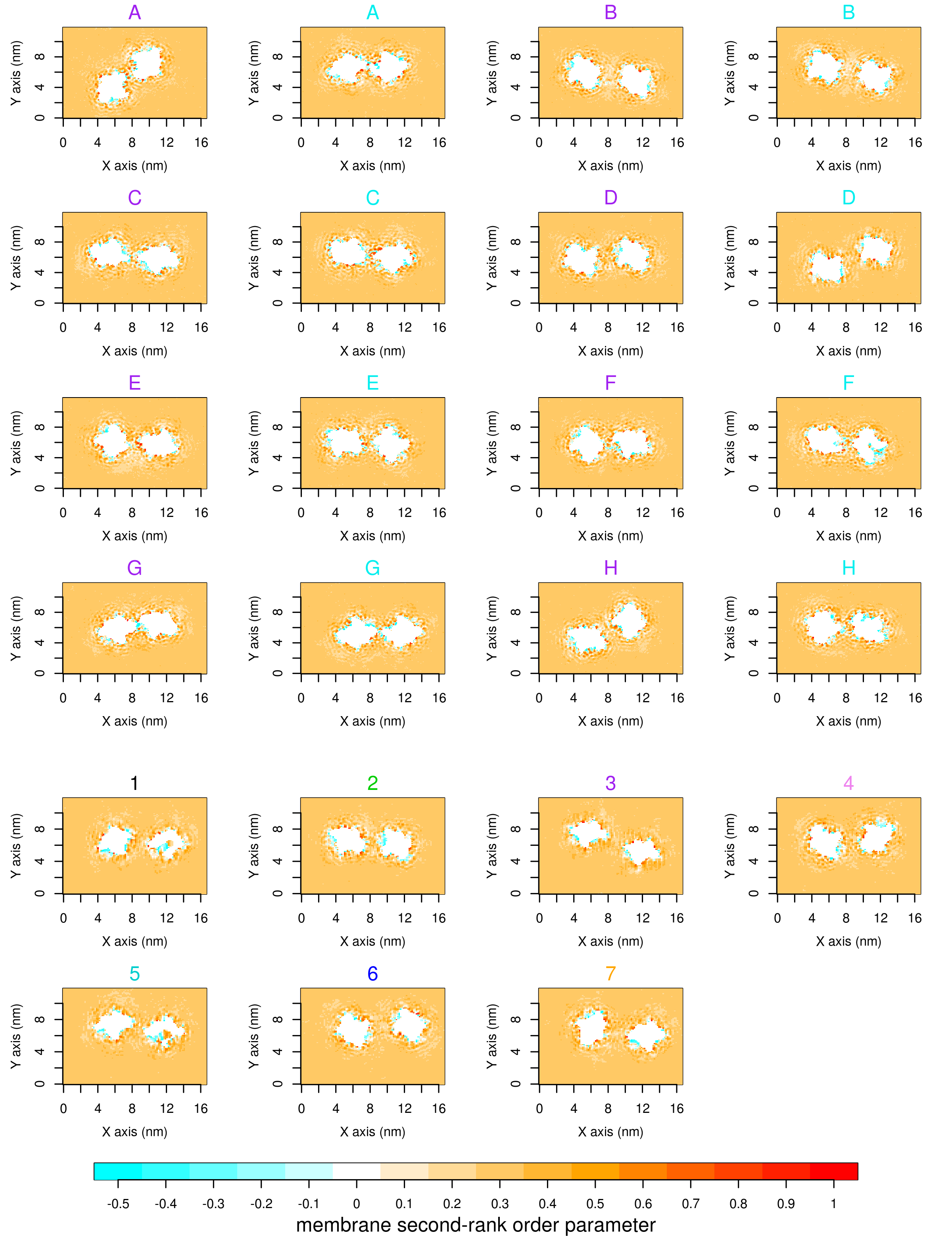

Supplement: S9 Fig — A-I) Membrane order parameter is shown for two representative systems per cluster. These are the same systems as used in the PMF calculations, labeled according to Fig 6 (cyan and magenta color of panel label). We use the trajectories of the first umbrella window and averaged the membrane order parameter over the second half of each trajectory. The starting structures of each simulation are the respective final structures of the DAFT simulations. The systems are shown from the intracellular side. The ordering of lipids is estimated using a second-rank order parameter defined as: S = 1/2 * (3 * <cos2(θ)> − 1), where θ is the angle between the membrane normal and the bond between two successive beads of the Martini lipid model. The brakets < > represent ensemble averaging. A value of 1 indicates that the lipids would be perfectly aligned with the axis, while -0.5 indicates an orientation parallel to the membrane plane. A decrease/increase in ordering of the annular lipids is visible in the annular structure surround hDAT. The lipids directly attached to hDAT show the strongest deviation from bulk order parameter. Panel 1–7 shows the membrane order parameter of the transient dimers coloured according to Figs 3B and 6I. Panels 1–7 do not indicate a pattern of specific deviation that would set these systems apart from the members of cluster A-H. This indicates that lipid ordering is not a driving force that prevents dimerization at the bundle domain. (TIF) [file pcbi.1006229.s009.tif]

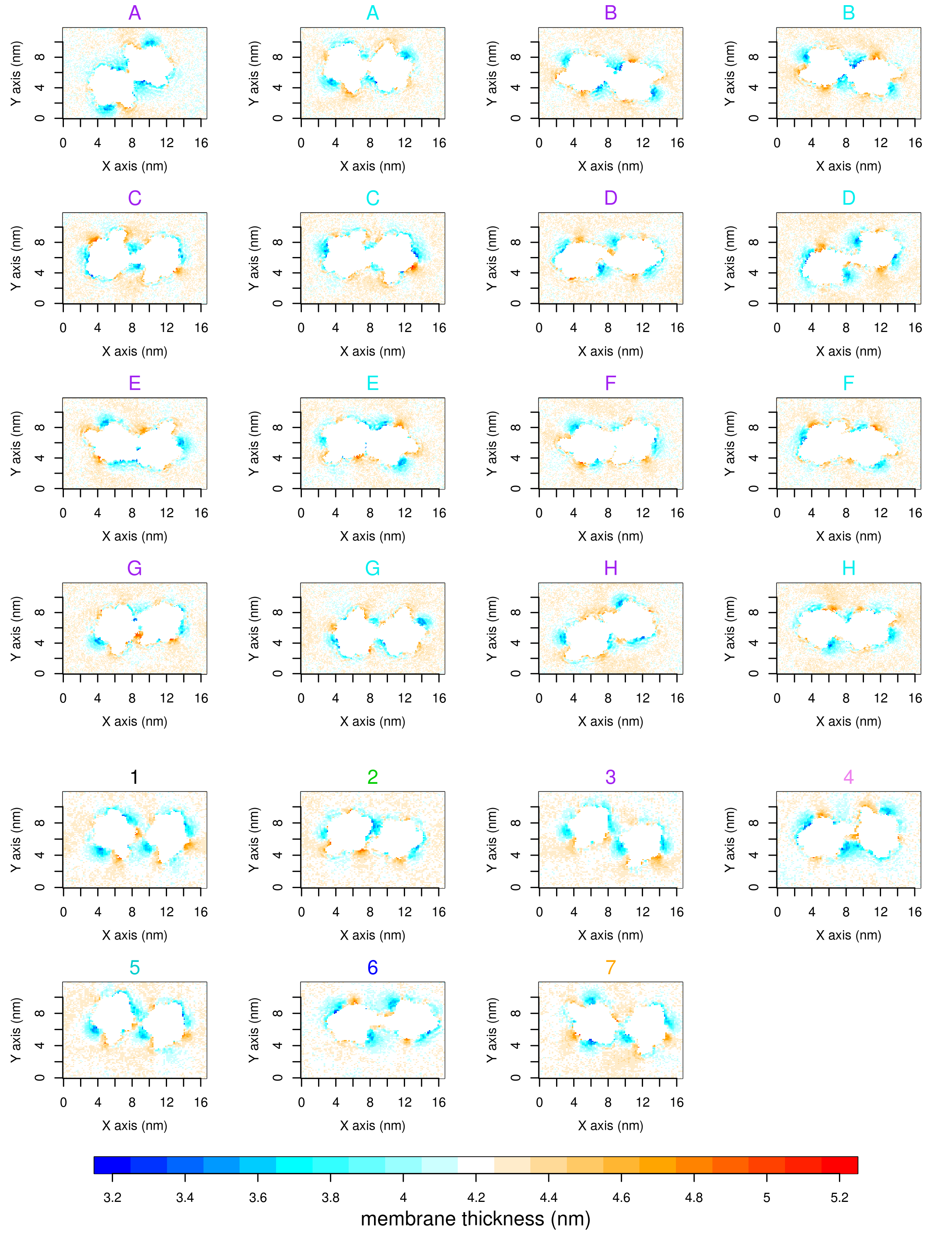

Supplement: S10 Fig — A-H) Membrane thickness is shown for two representative systems per cluster. These are the same systems as used in the PMF calculations, labelled according to Fig 6 (cyan and magenta color of panel label). We used the trajectories of the first umbrella window and averaged the membrane thickness over the second half of each trajectory. The starting structures of each simulation are the respective final structures of the DAFT simulations. The systems are shown from the intracellular side. Panel 1–7 show membrane thickness of the transient dimers coloured according to Figs 3B and 6I. Panels 1–7 show in most system a strong change of membrane thickness across the dimer interface, placing areas of high membrane thickness next to areas of low membrane thickness. Overall an area of increased membrane thickness at one protomer is paired with an area of low membrane thickness of the second protomer. The mismatch in pairing of membrane thickness might contribute to the inability to form dimers, which include the bundle domain. The same mismatch was not observed for the stable dimers from cluster A-H. (TIF) [file pcbi.1006229.s010.tif]

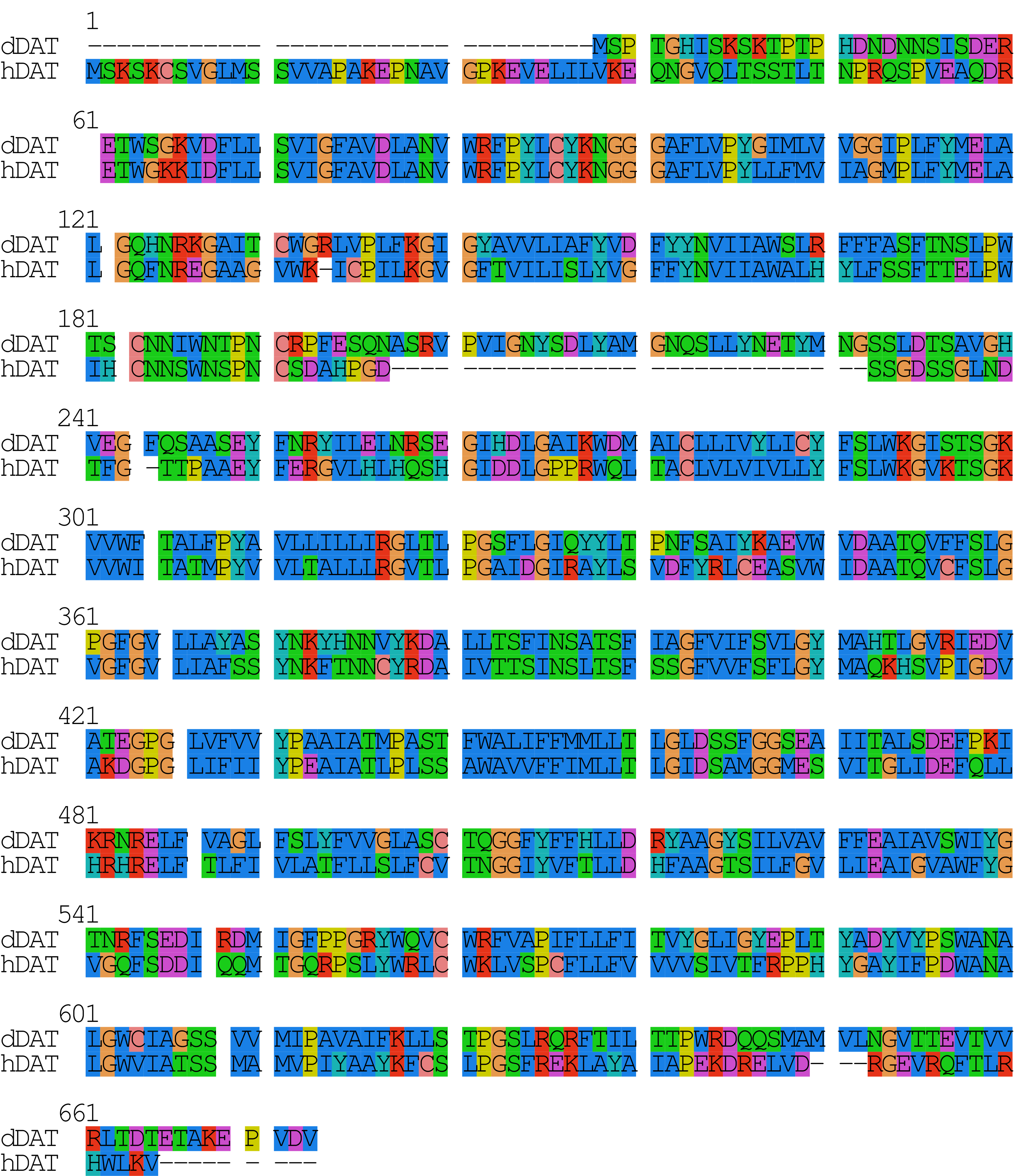

Supplement: S11 Fig — Sequence alignment of the dopamine transporters from human and Drosophila melanogaster. Residues are coloured according to the color code of clustal. (TIF) [file pcbi.1006229.s011.tif]

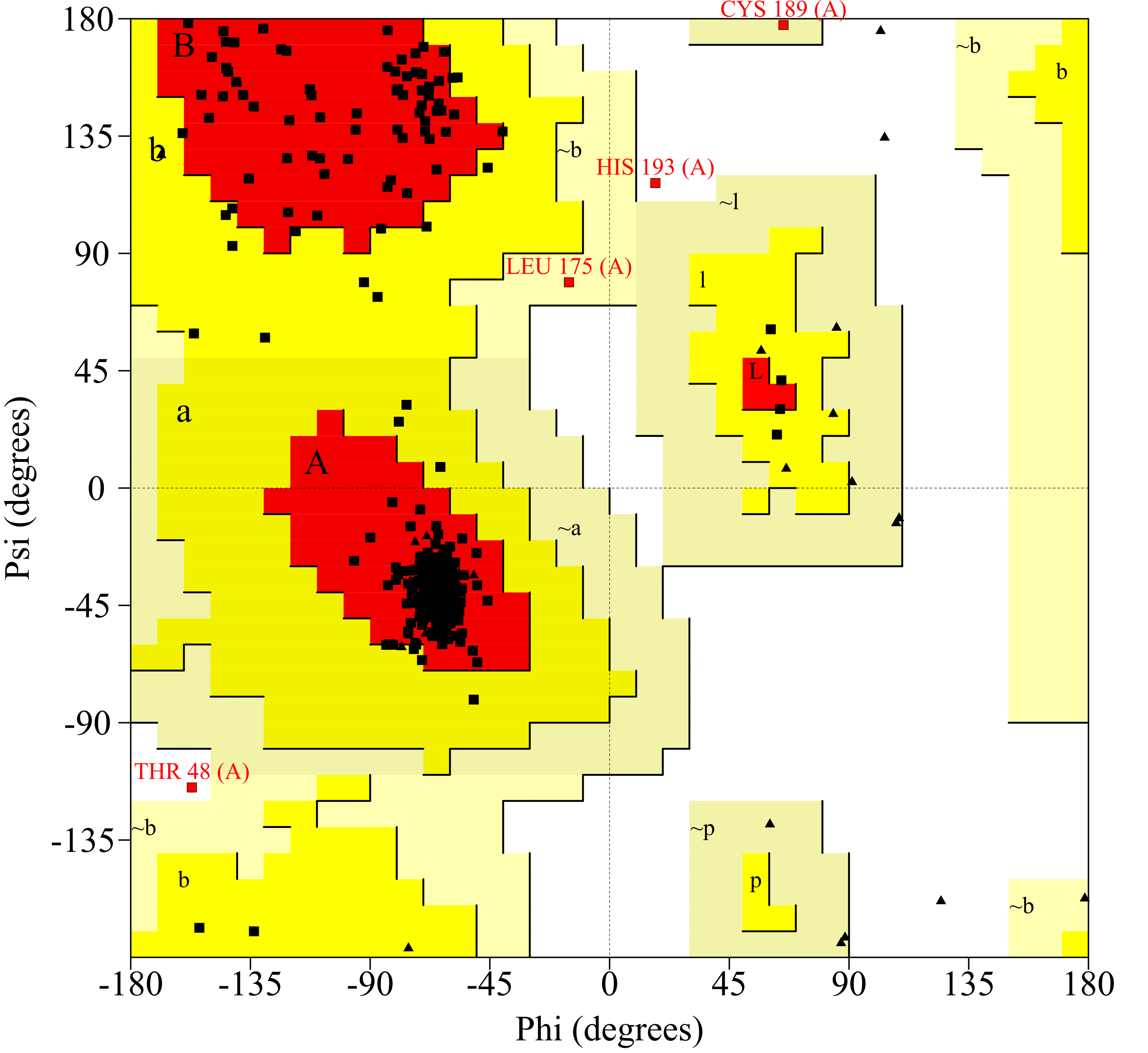

Supplement: S12 Fig — The Ramachandran plot represents a quality assessment of the backbone geometry of the hDAT model: 94.0% of residues are in the most favourable region, 5.2% in the additional allowed region, 0.4% in the generously allowed region, and 0.4% are found the disallowed region of the Ramachandran plot. (TIF) [file pcbi.1006229.s012.tif]

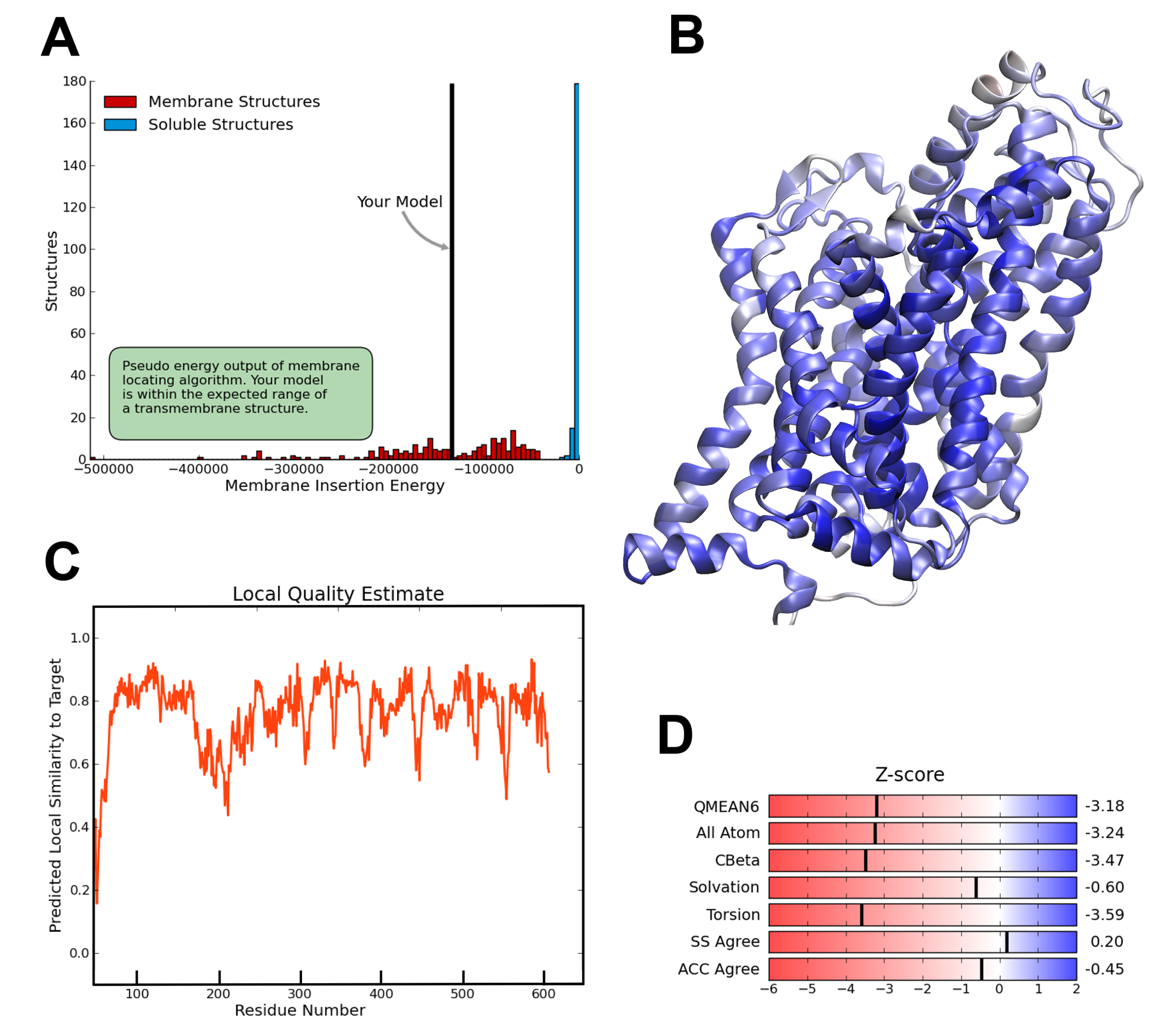

Supplement: S13 Fig — QMEAN is a scoring function that allows for assessing model quality using structural descriptors including local geometry, structural compactness, secondary structure, and solvation. The QMEANBrane score is specifically optimized for transmembrane proteins. A) Comparison of QMEANBrane score to reference dataset of membrane proteins. B) Mapping of the local QMEANBrane score on the model of hDAT, C) Per residues score showing per residue model quality. D) Z-score. (TIF) [file pcbi.1006229.s013.tif]
