# Supplementary material for: Dopamine transporter oligomerization involves the scaffold domain, but spares the bundle domain
Source: PLoS Comput Biol. 2018 Jun 6;14(6):e1006229. doi: 10.1371/journal.pcbi.1006229 (PMC6005636; doi:10.1371/journal.pcbi.1006229)
Supplement: S1 Table — Summary of the number of dimer per cluster at 2μs as observed in Fig 2. Both off-diagonal clusters were merged in the asymmetric dimers. (DOC) [file pcbi.1006229.s014.doc]

| Clusters | Number of dimers per cluster | Dimers (%) |
| --- | --- | --- |
| A | 9 | 1.8 |
| B | 42 | 8.2 |
| C | 11 | 2.1 |
| D | 25 | 4.9 |
| E | 44 | 8.6 |
| F | 58 | 11.3 |
| G | 31 | 6.1 |
| H | 27 | 5.3 |
