# Supplementary material for: Dopamine transporter oligomerization involves the scaffold domain, but spares the bundle domain
Source: PLoS Comput Biol. 2018 Jun 6;14(6):e1006229. doi: 10.1371/journal.pcbi.1006229 (PMC6005636; doi:10.1371/journal.pcbi.1006229)
Supplement: S2 Table — The PMF profiles shown in Fig 6 are integrated and the integrals reported. The energy at full separation are set to zero. The color code of the cluster column is consistent with Fig 6. (DOC) [file pcbi.1006229.s015.doc]

| **Cluster** | **Integral (kJ/mol/nm)** | **Standard deviation (kJ/mol/nm)** |
| --- | --- | --- |
| **A** | -866 | 295 |
| **A** | -1761 | 363 |
| **B** | -1198 | 204 |
| **B** | -2012 | 175 |
| **C** | -874 | 124 |
| **C** | -2111 | 208 |
| **D** | -349 | 198 |
| **D** | -818 | 209 |
| **E** | -1146 | 160 |
| **E** | -1650 | 298 |
| **F** | -1770 | 244 |
| **F** | -1699 | 282 |
| **G** | -1838 | 303 |
| **G** | -2087 | 201 |
| **H** | -1192 | 153 |
| **H** | -662 | 163 |
| **I1** | 243 | 139 |
| **I2** | -12 | 112 |
| **I3** | -148 | 105 |
| **I4** | -13 | 137 |
| **I5** | -494 | 120 |
| **I6** | -262 | 159 |
| **I7** | 331 | 131 |
